# Preparation and characterization of graphitic carbon nitride-supported l-arginine as a highly efficient and recyclable catalyst for the one-pot synthesis of condensation reactions

**DOI:** 10.1038/s41598-021-97360-x

**Published:** 2021-10-05

**Authors:** Hossein Ghafuri, Zeinab Tajik, Nastaran Ghanbari, Peyman Hanifehnejad

**Affiliations:** Catalysts and Organic Synthesis Research Laboratory, Department of Chemistry, Iran University of Science and Technology, 16846‑13114 Tehran, Iran

**Keywords:** Medical research, Chemistry

## Abstract

In this work, graphitic carbon nitride-supported l-arginine (g-C_3_N_4_@l-arginine) nanocatalyst was synthesized and evaluated using FT-IR, EDX, XRD, TGA, and FESEM analyses. The performance of the prepared nanocatalyst was examined in the synthesis of 1,4-dihydropyridine, 4H-chromene, and 2,3-dihydro quinazoline derivatives. The novel g-C_3_N_4_@l-arginine nanocatalyst showed high thermal stability, easy separation from reaction media, the capability to be used in various multicomponent reactions, and acceptable reusability.

## Introduction

These days, most chemical processes are carried out in the presence of catalysts. Among various catalysts, supported catalysts have more application than other catalysts. Synthesis of this class of catalysts requires a support with high surface area for the adequate dispersion of primary catalyst. For this reason, using a suitable support is of high importance in the synthesis of such catalysts^1–4^.

Graphitic carbon nitride (g-C_3_N_4_) can be used as a metal-free catalyst or catalyst support due to its excellent properties and exceptional performance. g-C_3_N_4_ sheets are an important class of conjugated polymers for the synthesis of new heterogeneous catalysts due to their unique electronic band structure, excellent physical, chemical, and thermal stability, high abrasion resistance, high hardness, low density, versatile performance, low synthesis cost, and recyclability ^5,6^. On the other hand, amino acids are one of the most interesting catalyst substrates due to their unique structure ^7^. Arginine is one of the main and semi-essential amino acids in the body of living organisms with advantages such as nontoxicity, ability to easily bind to catalytic support, and low cost for the preparation of acidic catalysts. In addition, acidic catalysts play an important role in the synthesis of organic compounds. Heterocyclic compounds are one of the best candidates in organic synthesis and pharmaceutical chemistry. They are mainly used as medicines, chemicals, veterinary products, disinfectants, expanders, and antioxidants ^8^.

The use of inexpensive and non-toxic reagents as well as low waste production is of great importance in green chemistry reactions. Hence, multicomponent reactions (MCRs) are considered as a useful method for the synthesis of heterocyclic organic molecules. Significant advantages of MCRs are the elimination of intermediates, short reaction times, high reaction yield, and easy separation of products ^9–12^. Compounds such as 1,4-dihydropyridine, tetrahydro-4*H*-chromenes, and dihydroquinazolines, which have high medicinal activity, are synthesized by MCRs.

Dihydropyridines are divided into two classes; symmetrical and asymmetrical. The latter is synthesized by the reaction of an aldehyde, 2 mmol of two different β-keto esters, and a nitrogen donor such as ammonium acetate or ammonia. The product of the initial reaction is dihydropyridine that can later be converted to pyridine. These compounds are an important class of antihypertensive drugs, vasodilators, hypnotic, anti-tumor ^13,14^, anti-inflammatory^15,16^, anti-diabetic, anti-anxiety, anti-mutation, and are known as calcium channel blockers^17^.

Tetrahydro-4*H*-chromenes are an important class of heterocyclic compounds with simple structure and low side effects. They are synthesized by the single-step condensation of aldehydes with malononitrile and dimedone^18^. In addition, their derivatives have important activities such as anticancer, antiviral, anti-inflammatory, antibacterial, antifungal, antioxidant, and anticoagulant. They are also used as cognitive enhancers to treat Alzheimer's disease^19,20^.

Dihydroquinazolines are the building blocks of about 150 natural alkaloids which are prepared by the reaction between aldehydes, isotonic anhydride, and ammonium acetate. Moreover, they have a range of pharmaceutical and biological activities such as anti-inflammatory, antimalarial, antibacterial, anticancer, and antiviral activities ^21,22^. Hence, many efforts have been made to synthesize such high-yield compounds. There are various methods for the synthesis of these compounds using different catalysts such as MCM-41@Schiff base-Co (OAC)_2_^23^, Yb (NPf_2_)_3_^24^, MCM-41@serine@Cu(II) ^25^, titanium silicon oxide nanopowder^26^, Y(NO_3_)_3_.6H_2_O ^27^, etc. despite their numerous advantages, they have some limitations such as long reaction time, expensive reagents, and the possibility of their contamination in final products.

**Figure 1:**
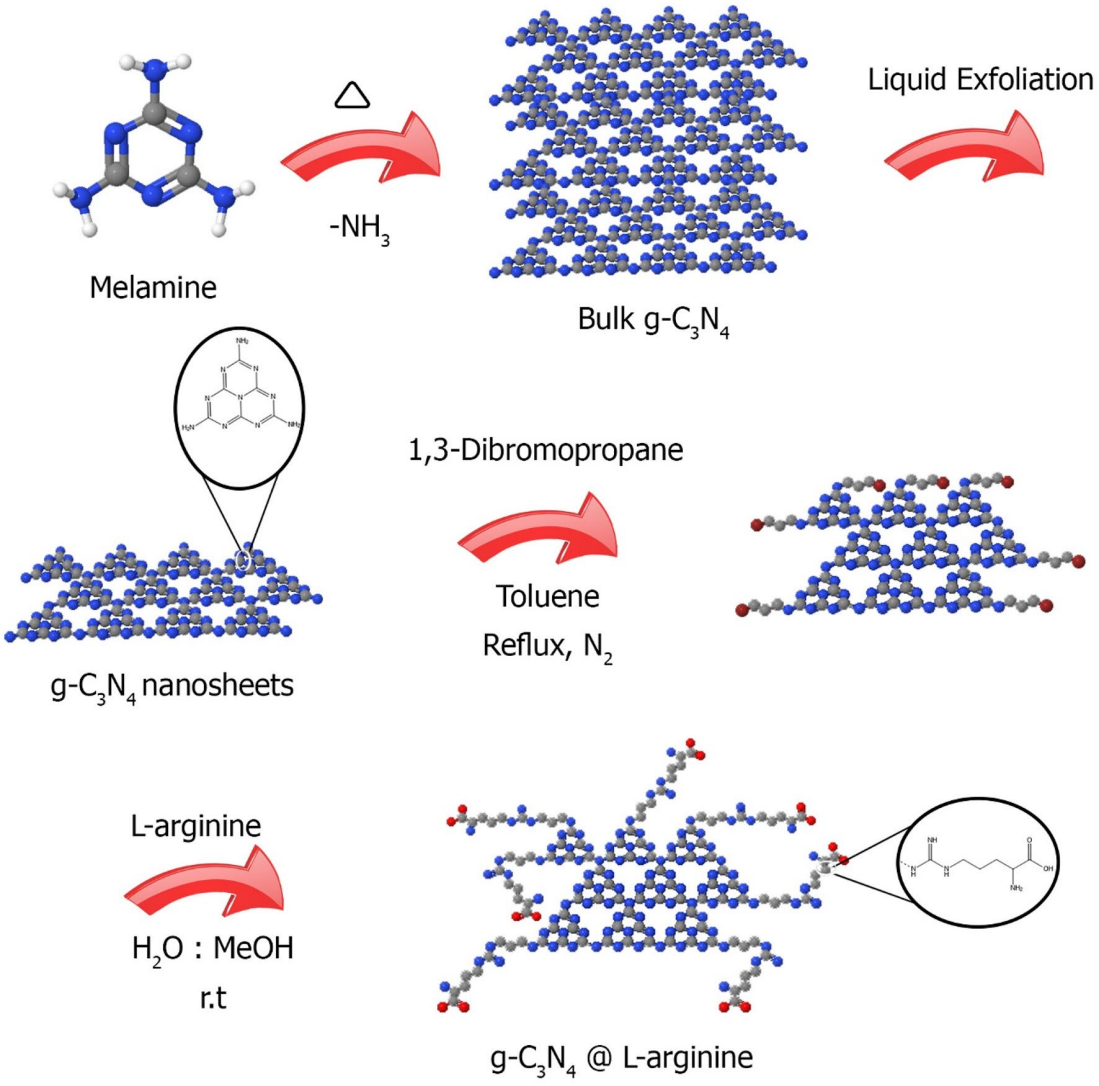
Synthesis of g-C_3_N_4_@l-arginine.

In this paper, new g-C_3_N_4_@l-arginine catalyst with the ability to perform various multi-combination reactions with high yield, short reaction time, recyclability, and easy separation from the reaction mixture is synthesized and examined (Fig. 1).

## Results and discussion

The g-C_3_N_4_@l-arginine synthesis process consists of three main steps, as shown in Fig. 1. The first step is the synthesis of nanosheet g-C_3_N_4_ from melamine, which melamine was polymerized to bulk g-C_3_N_4_, then nanosheet g-C_3_N_4_ is synthesized by liquid exfoliation and sonication. In the second step, g-C_3_N_4_ nanosheets were modified by 1,3-dibromopropane at 100 °C for 24 h under nitrogen atmosphere. Finally, the g-C_3_N_4_@l-arginine was obtained via the reaction between l-arginine and modified g-C_3_N_4_ nanosheets. In this work, various techniques such as FTIR, EDX, XRD, FESEM, and TGA have been used to identify and characterize the novel nanocatalyst.

**Figure 2:**
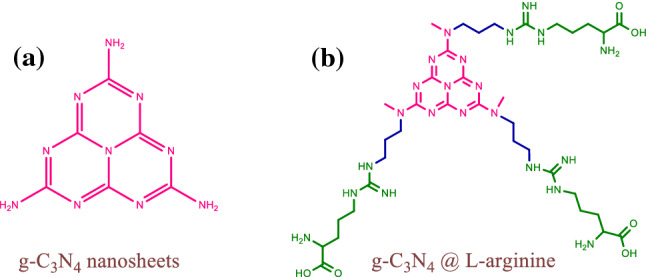
Nanosheets g-C_3_N_4_, (**a**) and g-C_3_N_4_@l-arginine (**b**).

**Figure 3:**
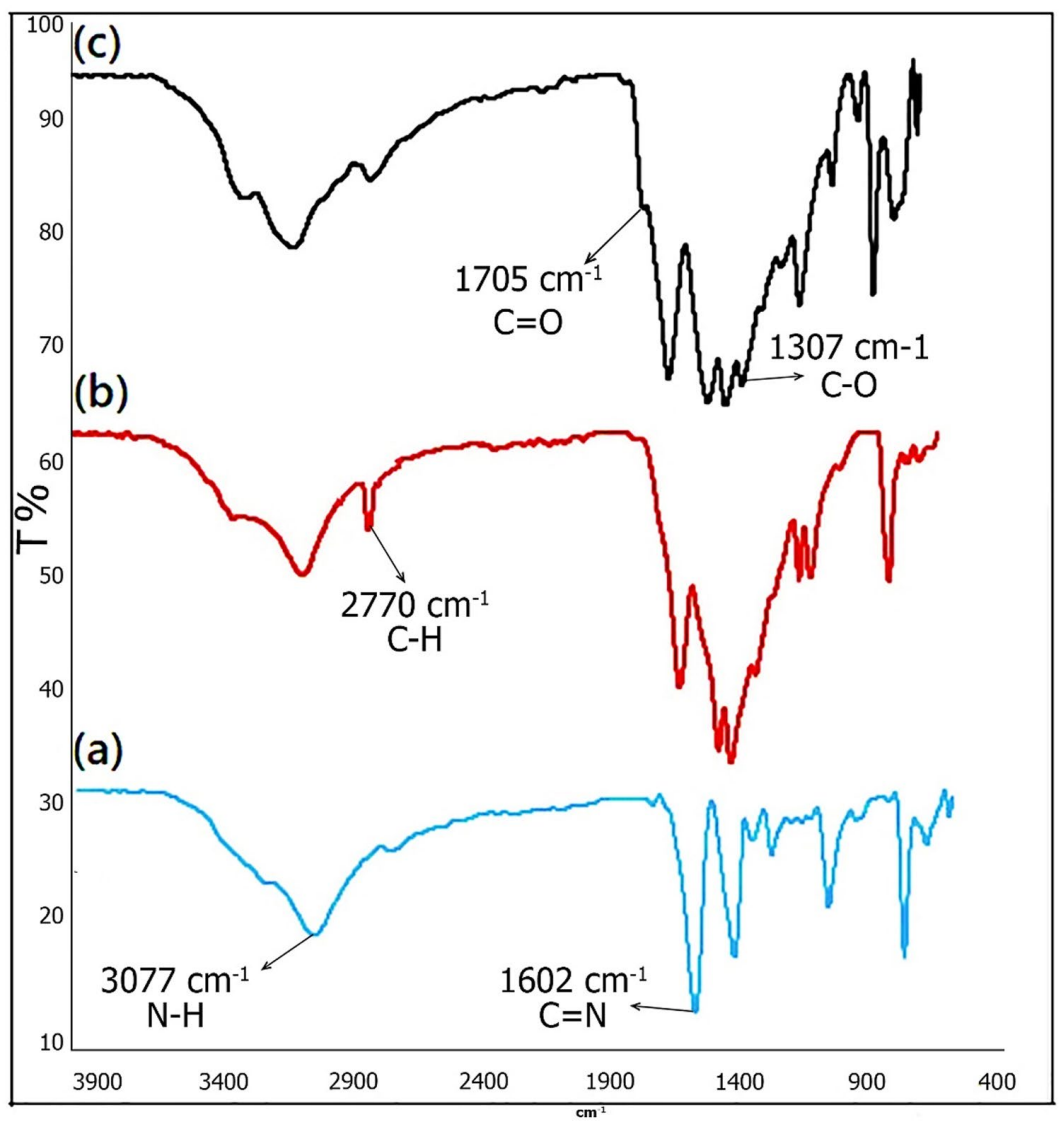
FT-IR spectra of (**a**) nanosheets g-C_3_N_4_, (**b**) modified g-C_3_N_4_, (**c**) g-C_3_N_4_@l-arginine.

FTIR spectra of g-C_3_N_4_ nanosheets (Fig. 2a) and g-C_3_N_4_@l-arginine nanocatalyst (Fig. 2b) are shown in Fig. 2. The strong and broad peak in the range of 3000–3300 cm^−1^ is related to stretching vibration of N–H bonds, breadth peak can be assigned to N–H groups involved in H-bonding or the presence of O–H groups due to water adsorption by nanosheets g-C_3_N_4_^28,29^. The stretching vibration peak of C=N can be observed at 1602 cm^−1^. The peaks at 1303 and 1082 cm^−1^ are attributed to the stretching vibration of C–N bonds formed between triazine and N–H groups, while the stretching vibration of C–N bonds in the ring is easily visible at 1448 and 1379 cm^−1^ ^29,30^. In addition, the peak at 786 cm^−1^ is associated with the vibration of tri-s-triazine units ^1^ (Fig. 3a). Figure 3b shows that the g-C_3_N_4_ nanosheets has been modified with 1,3-dibromopropane; the peak at 3000–2800 cm^−1^ is related to C-H stretching vibration.

The spectrum of g-C_3_N_4_@l-arginine is presented in Fig. 3c in which the existence of l-arginine on the surface of g-C_3_N_4_ nanosheets can be confirmed based on the 1705 cm^−1^ and 1307 cm^−1^ peaks relating to the stretching vibration of C = O and C-O bonds, respectively. O–H and C-H bonds already existed in the structure of modified nanosheets g-C_3_N_4_.

**Figure 4:**
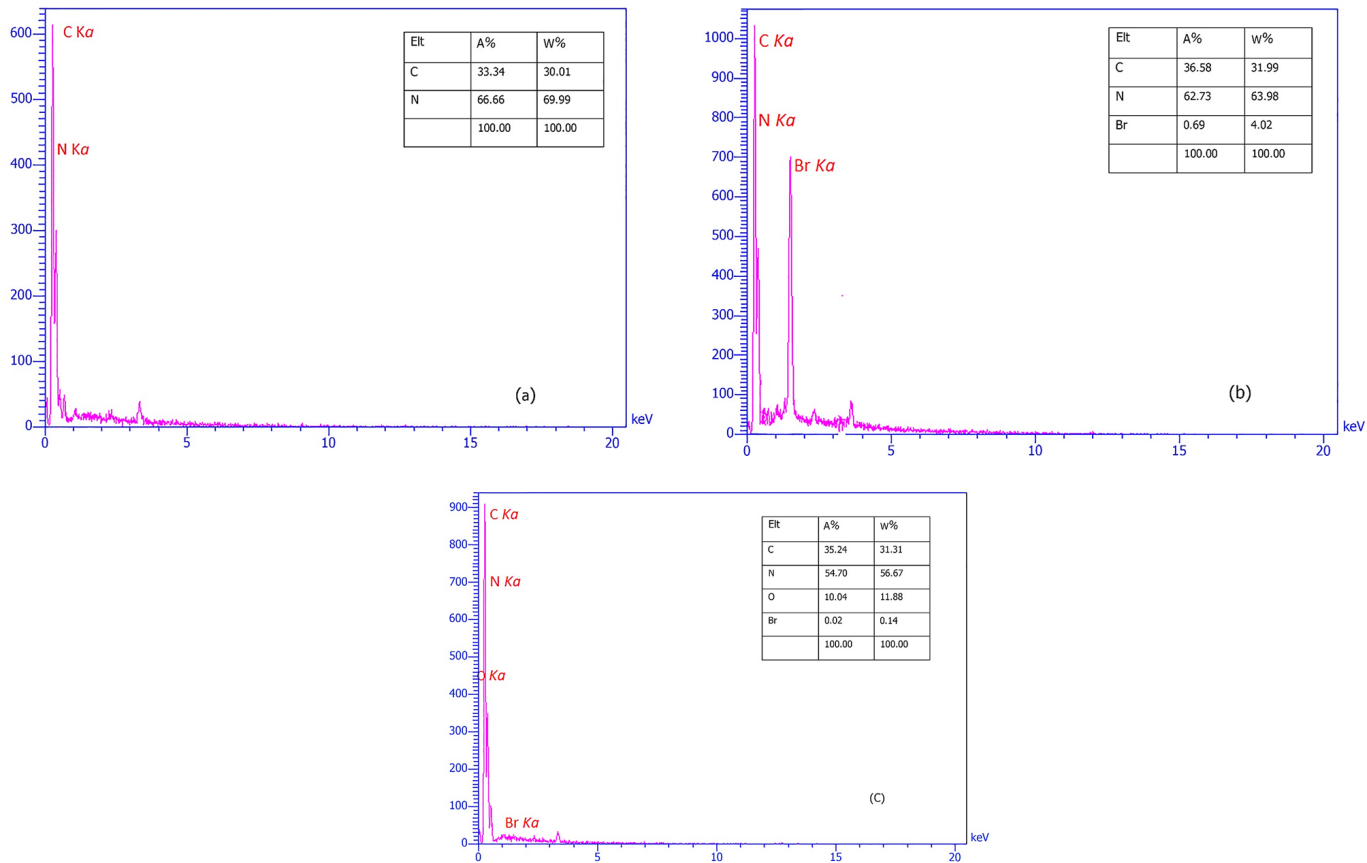
EDX spectrum of **(a**) nanosheets g-C_3_N_4_, (**b**) modified g-C_3_N_4_, (**c**) g-C_3_N_4_@l-arginine.

The presence of carbon and nitrogen elements in the structure of g-C_3_N_4_ nanosheets is visible in Fig. 4a. The presence of Br element in the structure proves that g-C_3_N_4_ nanosheets have been modified by 1,3-dibromopropane (Fig. 4b). Finally, the presence of carbon, nitrogen, and oxygen in the final structure (g-C_3_N_4_@l-arginine) confirmed the synthesis of g-C_3_N_4_@l-arginine nanocatalyst (Fig. 4c).

**Figure 5:**
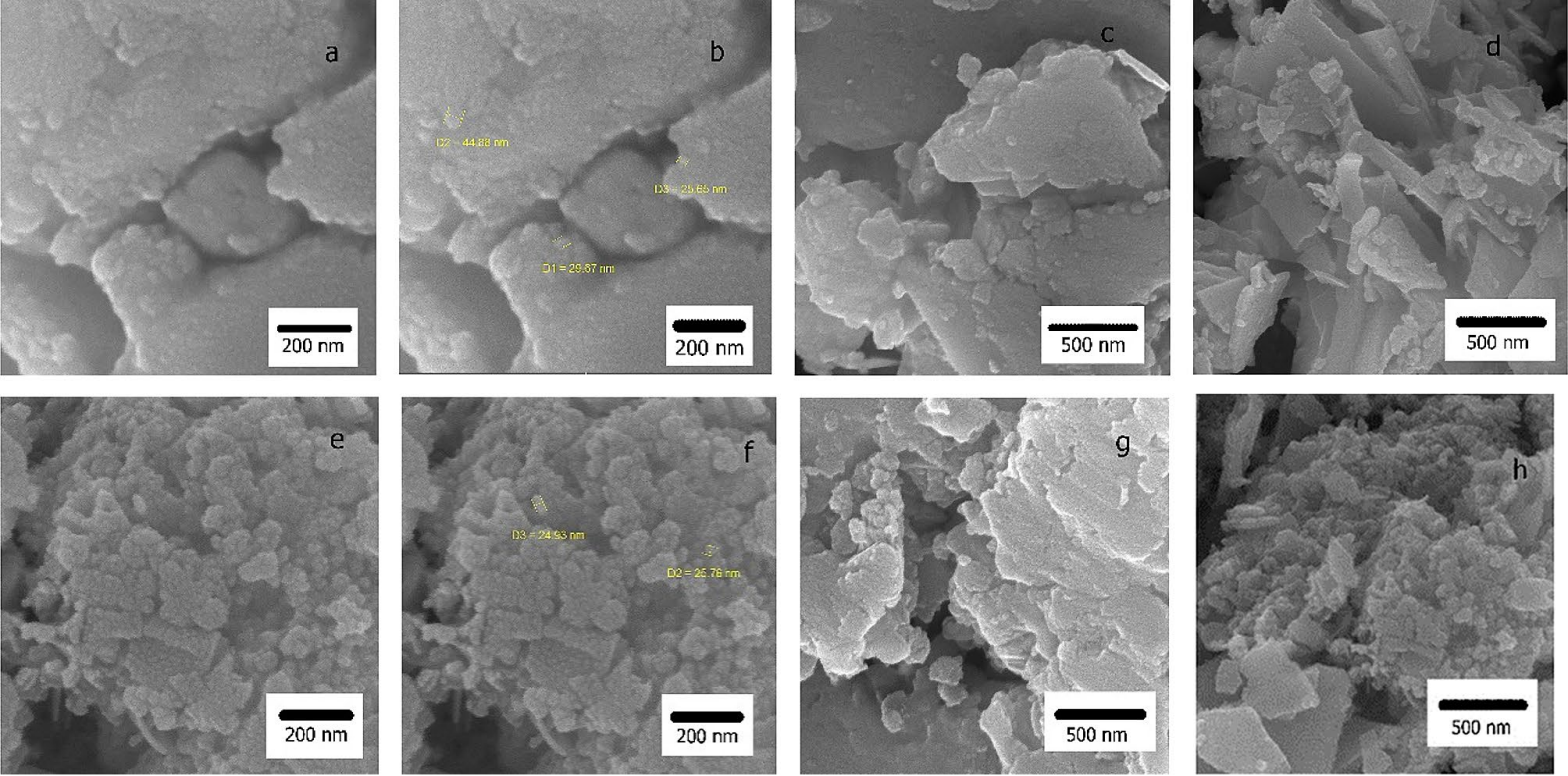
FE-SEM image of nanosheets g-C_3_N_4_ (**a**, **b**, **c**, and **d**), and g-C_3_N_4_@l-arginine (**e**, **f**, **g,** **h**).

The morphology of g-C_3_N_4_ nanosheets and g-C_3_N_4_@l-arginine was investigated by FE-SEM. Figure 5a-d shows FE-SEM images of g-C_3_N_4_ nanosheets. As shown in Figs. 5a-c, the g-C_3_N_4_ nanosheets have a smooth and flat surface, while in Fig. 5d, g-C3N4 nanosheets are irregular and connected together. FE-SEM images of g-C_3_N_4_@l-arginine are shown in Fig. 5e-h. It can be seen from Fig. 5e-g that g-C_3_N_4_ nanosheets have a flake-like morphology with a relatively rough surface, mainly due to the presence of l-arginine on the surface of g-C_3_N_4_ nanosheets. In Fig. 5h, more irregular-shape g-C_3_N_4_ nanosheets with tiny particles on the surface are observed, which again confirms the deposition of l-arginine on the g-C_3_N_4_ nanosheets.

**Figure 6:**
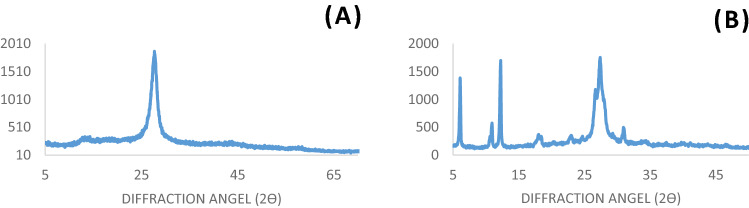
XRD pattern of (**a**) nanosheets g-C_3_N_4_, (**b**) g-C_3_N_4_@l-arginine.

XRD patterns of g-C_3_N_4_ nanosheets and g-C_3_N_4_@l-arginine are shown in Figs. 6a and 4b, respectively. The diffraction peaks at 2θ = 27.69 and 15.96 (Fig. 6a) prove the successful synthesis of g-C_3_N_4_ nanosheets ^1,7,28^, while the diffraction peaks at 2θ = 6.07, 10.85, 12.21, 23.60, and 30.97 (Fig. 6b) correspond to l-arginine (JCPDS card no. 00–004-0180), confirming the presence of l-arginine on the surface of g-C_3_N_4_ nanosheets.

**Figure 7:**
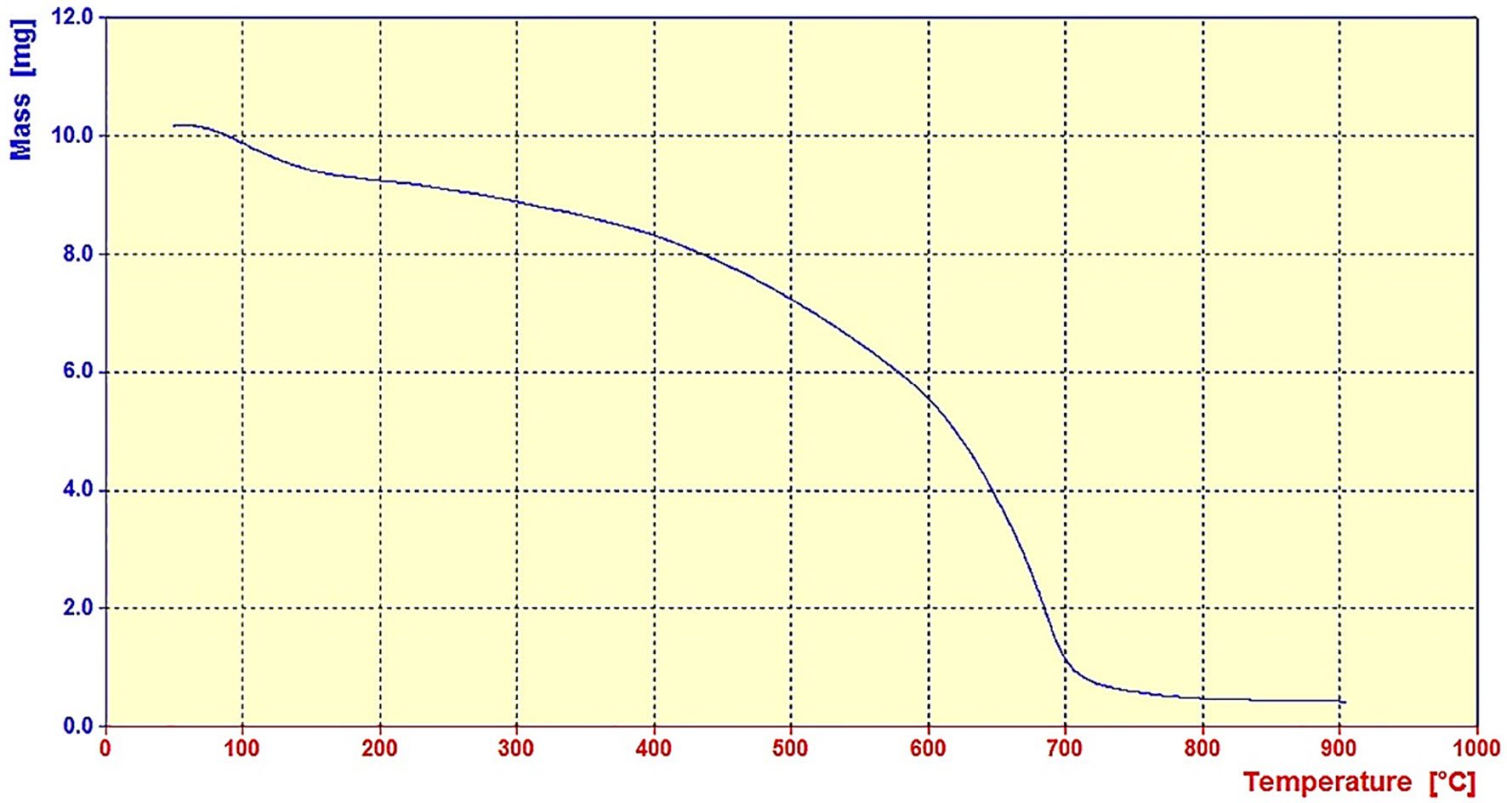
TGA analysis of g-C_3_N_4_@l-arginine.

Figure 7 shows the thermal stability of the synthesized g-C_3_N_4_@l-arginine in the range of 50–800 °C. As can be seen, the weight ratio has gradually decreased by increasing the temperature from 100 to 200 °C, which is most likely related to the removal of water absorbed on the surface of g-C_3_N_4_@l-arginine. Then, another weight loss is observed in the range of 200 to 400 °C, which is attributed to the separation of l-arginine from the structure. Finally, there is another weight loss in the range of 400 to 700 °C due to the decomposition of g-C_3_N_4_ nanosheets ^31^.

### Model reactions

The performance of the prepared g-C_3_N_4_@l-arginine nanocatalyst was evaluated for the synthesis of 1,4-dihydropyridine, 4*H*-chromene, and 2,3-dihydro quinazoline derivatives. For this purpose, various parameters such as reaction time, catalyst concentration, and the solvent were examined (Table 1). The reaction of 4-chlorobenzaldehyde (1 mmol), ethyl acetoacetate (1 mmol), dimedone (1 mmol), and ammonium acetate (1 mmol) for the synthesis of 1,4-dihydropyridine derivatives, the reaction of 4-chlorobenzaldehyde (1 mmol), dimedone (1 mmol), and malononitrile (1 mmol) for the synthesis of 4*H*-chromene derivatives, and the reaction of 4-chlorobenzaldehyde (1 mmol), isotonic anhydride (1 mmol), and ammonium acetate (1 mmol) for the synthesis of 2,3-dihydro quinazoline derivatives were considered as model reactions with and without g-C_3_N_4_@l-arginine nanocatalyst under different conditions. The reaction progress was monitored by Thin-layer chromatography (TLC). As can be seen in Table 1 (entries 1 and 2), no progress was observed for the model reactions without nanocatalyst. By introducing 1.00 mg of g-C_3_N_4_@l-arginine (Table 1, entry 3), however, the model reactions occurred easily. Then, the influence of other parameters including catalyst concentration, reaction time, and solvent were examined. As can be seen, time had not significant effect on the reaction progress, thus 15 min was considered as the optimum reaction time for all the model reactions (Table 1, entries 7, 8, and 9). Furthermore, the highest product yield was obtained using ethanol as solvent at 80 °C in the presence of 20.00 mg of g-C_3_N_4_@l-arginine (Table 1, entry 5).

**Table 1:** Optimization of different parameters for model reactions 1 to 3.

Entry	Catalyst	Catalyst loading (mg)	Solvent	Time (min)	Temperature (°C)	Yield (%)		
						Reaction (1)^a^	Reaction (2)^b^	Reaction (3)^c^
1	–	–	EtOH	60	r.t	–	–	–
2	–	–	EtOH	60	Reflux	–	–	–
3	g-C_3_N_4_@l-arginine	1.00	EtOH	15	Reflux	53	60	57
4	g-C_3_N_4_@l-arginine	10.00	EtOH	15	Reflux	85	90	87
5	g-C_3_N_4_@l-arginine	20.00	EtOH	15	Reflux	94	97	96
6	g-C_3_N_4_@l-arginine	30.00	EtOH	15	Reflux	95	98	96
7	g-C_3_N_4_@l-arginine	20.00	EtOH	10	Reflux	89	97	93
8	g-C_3_N_4_@l-arginine	20.00	EtOH	20	Reflux	95	98	93
9	g-C_3_N_4_@l-arginine	20.00	EtOH	30	Reflux	96	98	94
10	g-C_3_N_4_@l-arginine	20.00	CH_2_Cl_2_	15	Reflux	54	67	60
11	g-C_3_N_4_@l-arginine	20.00	DMF	15	Reflux	52	56	55
12	g-C_3_N_4_@l-arginine	20.00	H_2_O	15	Reflux	75	78	77
13	g-C_3_N_4_@l-arginine	20.00	EtOH	15	r.t	57	66	68

^a^Reaction of 4-chlorobenzaldehyde (1 mmol), ethyl acetoacetate (1 mmol), dimedone (1 mmol), and ammonium acetate (1 mmol) for the synthesis of 1,4-dihydropyridine**.**

^b^Reaction of 4-chlorobenzaldehyde (1 mmol), dimedone (1 mmol), and malononitrile (1 mmol) for the synthesis of 4H-chromene.

^c^Reaction of 4-chlorobenzaldehyde (1 mmol), isotonic anhydride (1 mmol), and ammonium acetate (1 mmol) for the synthesis of 2,3-dihydroquinazoline.

In the following, various aldehydes were applied for the synthesis of 1,4-dihydropyridine, 4H-chromene, and 2,3-dihydro quinazoline derivatives under optimal reaction conditions. Based on model reactions that are provided in Tables 2, 3 and 4, a wide range of different derivatives of the desired multicomponent reactions were prepared with high yield.

**Table 2:** Synthesis of 1,4-dihydropyridine derivatives using g-C_3_N_4_@l-arginine nanocatalyst.


Entry	R	Product	Time (min)	Mp (°C)	Mp (°C, ref.)	Yield (%)
1	H	**5a**	10	217–219	218–220 ^17^	95
2	4-Cl	**5b**	15	240–242	241–243 ^17^	94
3	4-OH	**5c**	20	230–232	231–232 ^14^	89
4	4-NO_2_	**5d**	15	239–241	240–242 ^14^	90
5	4-Me	**5e**	25	254–256	250 ^32^	87

**Reaction conditions:** benzaldehyde (1 mmol), ethyl acetoacetate (1 mmol), dimedone (1 mmol), and ammonium acetate (1 mmol), g-C_3_N_4_@l-arginine (20 mg) and ethanol (7 mL) under reflux conditions.

**Table 3:** Synthesis of 4*H*-chromene derivatives using g-C_3_N_4_@l-arginine nanocatalyst.


Entry	R	Product	Time (min)	Mp (°C)	Mp (°C, ref.)	Yield (%)
1	H	**9a**	7	228–229	225–228 ^33^	95
2	4-Cl	**9b**	10	210–212	211–213 ^33^	97
3	4-NO_2_	**9c**	15	178–179	177–179 ^34^	88
4	2,4-Cl	**9d**	10	118–120	115–117 ^24^	95
5	4-OH	**9e**	20	206–208	208–210 ^24^	93
6	4-Me	**9f.**	30	217–220	220–221 ^35^	91
7	3-NO_2_	**9g**	20	206–207	206–209 ^34^	93
8	2-Cl	**9h**	15	213–214	211–213 ^35^	96
9	4-CN	**9i**	25	184–187	184–186 ^35^	89
10	4-OMe	**9j**	30	203–204	196–198^33^	87

**Reaction conditions:** Reaction of benzaldehyde (1 mmol), dimedone (1 mmol), and malononitrile (1 mmol) g-C_3_N_4_@l-arginine (20 mg) and ethanol (7 mL) under reflux conditions.

**Table 4:** Synthesis of 2,3-dihydroquinazoline derivatives using g-C_3_N_4_@l-arginine nanocatalyst.


Entry	R	Product	Time (min)	Mp (°C)	MP (°C , ref.)	Yield
1	H	**13a**	15	207–210	208–210^21^	90
2	4-Cl	**13b**	15	203–206	203–206^36^	96
3	3-NO_2_	**13c**	30	192–193	190–193^21^	89
4	4-OH	**13d**	25	213–215	213–215^36^	87
5	2-Cl	**13e**	15	205–206	206–208^22^	95
6	4-Me	**13f.**	25	200–201	198–201^22^	86
7	4-NO_2_	**13g**	20	201–202	200–202^36^	90
8	4-OMe	**13h**	25	180–182	178–182^36^	87
9	2,4-Cl	**13i**	35	163–166	165–169^21^	94
10	3-OH	**13j**	30	212–216	215–217^36^	89

**Reaction conditions:** benzaldehyde (1 mmol), isotonic anhydride (1 mmol), and ammonium acetate (1 mmol), C_3_N_4_@l-arginine (20 mg) and ethanol (7 mL) under reflux conditions.

### Reusability of g-C_3_N_4_@l-arginine nanocatalyst

**Figure 8:**
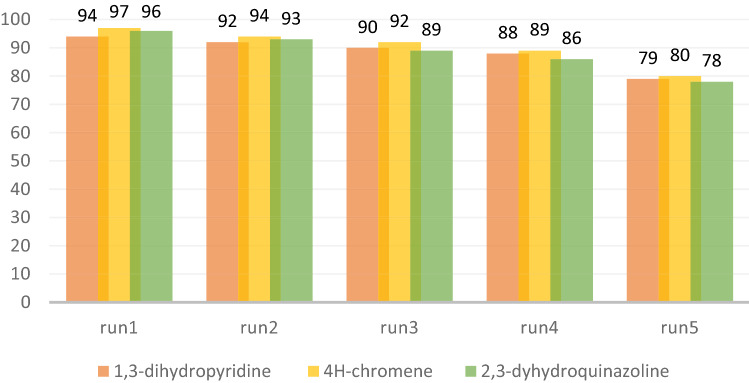
Examination of g-C_3_N_4_@l-arginine reusability in synthesis of 1, 3-dihydropyridine **5b,** 4*H*-chromene **9b**, and 2, 3-dihydroquinazoline **13b**.

**Figure 9:**
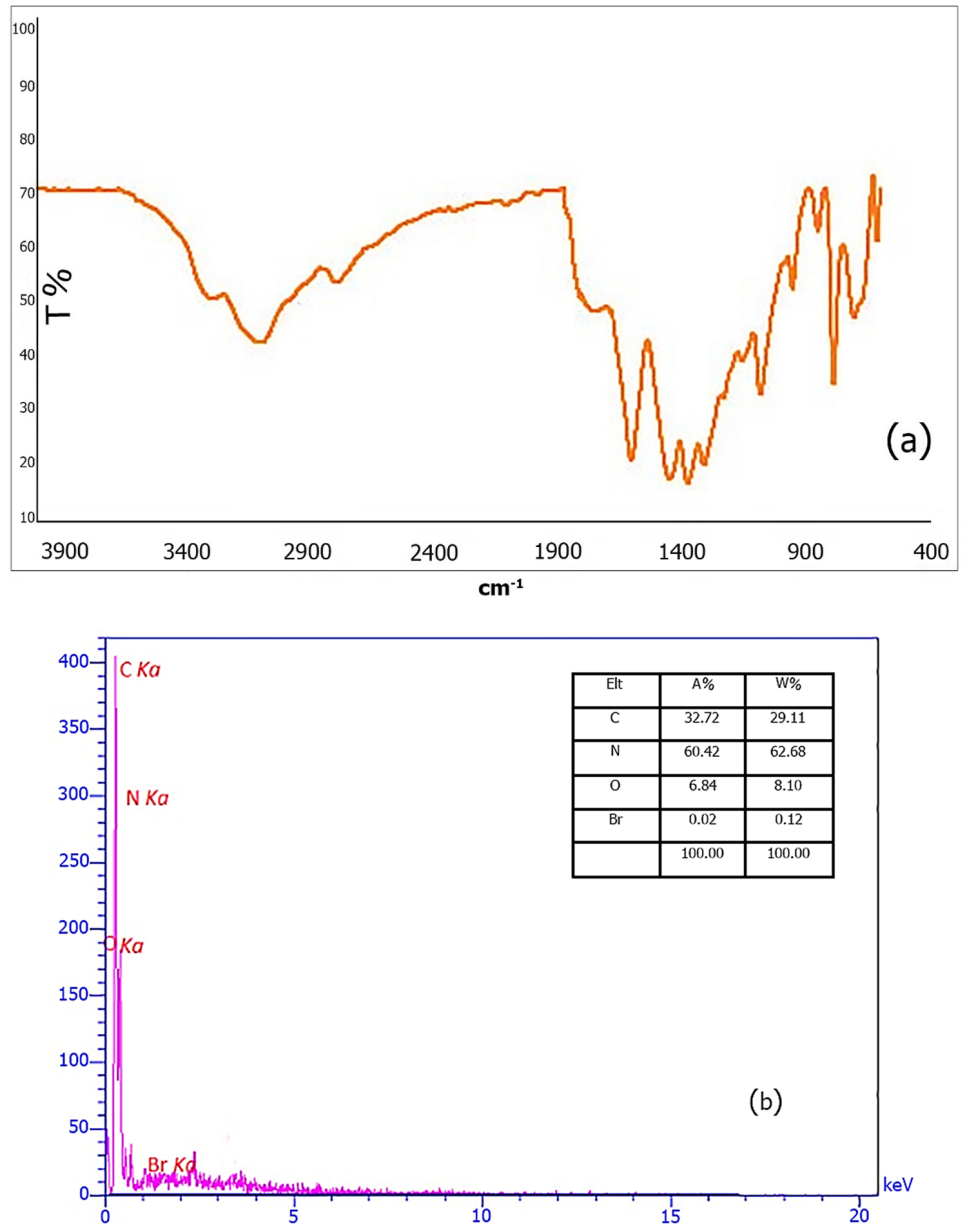
EDX (**b**) and FT-IR spectra (**a**) of g-C_3_N_4_@l-arginine after the five-times recycling.

According to the importance of recovery and recyclability in green chemistry, in this section, the reusability of g-C_3_N_4_@l-arginine was examined for the synthesis of 1,3-dihydropyridine **5b,** 4*H*-chromene **9b**, and 2,3-dihydroquinazoline **13b** products. For this purpose, after the first reaction (run 1), g-C_3_N_4_@l-arginine catalyst was separated from the reaction media, washed with ethanol, and dried in an oven at 70 °C. Then, the catalyst was reused for the next run. This was repeated for five times and the obtained yields were acceptable for catalytic reactions, and although performed reaction yields were decreased at each run bit by bit, in run 5th, the observed decrement was impressive in comparison with other runs (Fig. 8). EDX and FTIR spectra after the 5th run showed no significant changes in the primary structure of the g-C_3_N_4_@l-arginine nanocatalyst, as shown in Fig. 9.

### Mechanistic study of the prepared nanocatalyst in the synthesis of 1,4-dihydropyridine, 4*H*-chromene, and 2,3-dihydro quinazoline derivatives

**Figure 10:**
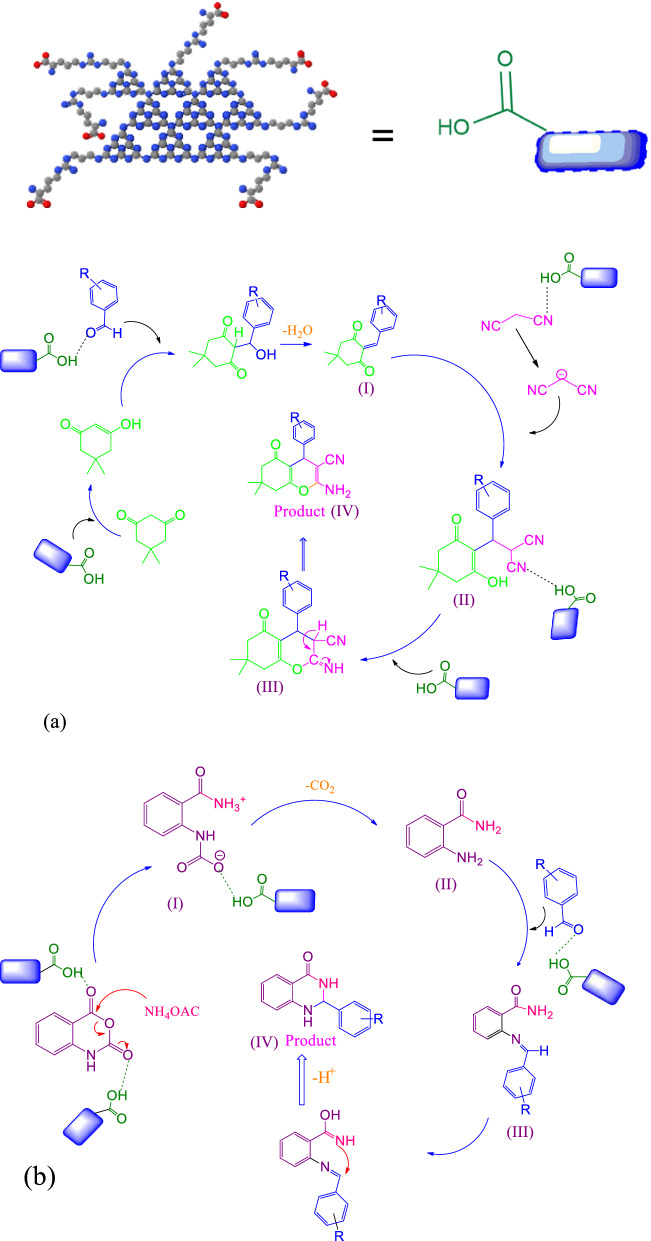

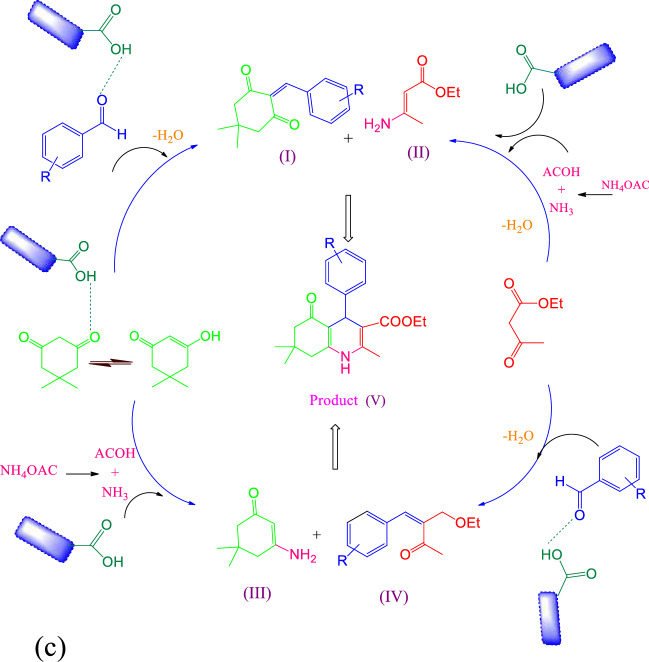
Proposed mechanism for synthesis of 4*H*-chromene derivatives (**a**), 2,3-dihydro quinazoline (**b**), and 1,4-dihydropyridine (**c**) by using g-C_3_N_4_@l-arginine.

In Fig. 10, the suitable mechanism for the formation of 1,4-dihydropyridine, 2,3-dihydro quinazoline, and 4*H*-chromene derivatives are provided. In each reaction, the presence of g-C_3_N_4_@l-arginine can activate reactants and different intermediates. As can be seen in Fig. 10a, 1,4-dihydropyridine derivatives can be synthesized in two methods. In the first method, aldehyde and dimedone produce intermediate** I** in the presence of g-C_3_N_4_@l-arginine, and the intermediate **II** is formed from the reaction between ethyl acetoacetate and ammonium acetate. But in the second method, dimedone and ammonium acetate produce intermediate **III** in the presence of g-C_3_N_4_@l-arginine, and the reaction between ethyl acetoacetate and aldehyde forms intermediate **IV**. Both methods ultimately lead to the formation of product **V**.

A suggested mechanism for the formation of 2,3-dihydro quinazoline derivatives is shown in Fig. 10b. At first, isotonic anhydride reacts with ammonium acetate in the presence of g-C_3_N_4_@l-arginine and produces intermediate **I**, then aldehyde activates by g-C_3_N_4_@l-arginine and adds to intermediate II. Finally, after removing H, the desired product **IV** is synthesized.

Figure 10c presents a probable method for the synthesis of 4*H*-chromene derivatives in the presence of g-C_3_N_4_@l-arginine is. In this mechanism, intermediate **I** is produced from the reaction between aldehyde and dimedone. Then, addition of malononitrile leads to the formation of intermediate **II**. At last, product **IV** is obtained.

### Catalytic activity of the synthesized nanocatalyst

Tables 5, 6 and 7 show the performance of g-C_3_N_4_@l-arginine in comparison with the catalysts reported in the literature for the synthesis of 1,4-dihydropyridine, 4H-chromene, and 2,3-dihydro quinazoline derivatives. For this purpose, various parameters such as catalyst concentration, reaction time, reaction temperature, and reaction yield were investigated. According to the data presented in each table, g-C_3_N_4_@l-arginine can be considered as a unique heterogonous nanocatalyst that can be used in a wide range of condensation reactions in addition to simple separation conditions of the reaction mixture. On the other hand, this nanocatalyst exceptionally showed higher synthesis yield at shorter reaction times.

**Table 5:** Comparison of catalytic activity of g-C_3_N_4_@l-arginine with other reported catalysts for the synthesis of *4H*-chromene derivatives.

Entry	Catalyst	Solvent/temperature	Time (min)	Chromene yield (%)	Ref
1	Fe_3_O_4_@MCM-41@Zr-piperazine-MNPs	EtOH/H2O/75 °C	40	74	^37^
2	AIL@MNP	Solvent-free/90 °C	25	89	^38^
3	MCM-41@Schiff base-Co(OAC)_2_	H_2_O/50 °C	180	94	^23^
4	Yb(NPf_2_)_3_	EtOH/80 °C	240	91	^24^
5	g-C_3_N_4_@l-arginine	EtOH/reflux	7	95	This work

Reaction conditions: benzaldehyde (1 mmol), dimedone (1 mmol), malononitrile (1.5 mmol), g-C_3_N_4_@l-arginine catalyst (20.00 mg), and ethanol (7 mL) under reflux.

**Table 6:** Comparison of catalytic activity of g-C_3_N_4_@l-arginine with other reported catalysts for the synthesis of 1,4-dihydropyridine derivatives^.^

Entry	Catalyst	Solvent/temperature	Time (min)	Hanztsch yield (%)	Ref
1	BNPs @ Si(CH2)3@NH SO3H	EtOH/reflux	25	95	^39^
2	Aluminized polyborate	Solvent-free/100 °C	15	94	^14^
3	Cell-Pr-NHSO_3_H	EtOH/reflux	45	91	^40^
4	MCM-41@serine@Cu(II)	EtOH/80 °C	170	96	^25^
5	g-C_3_N_4_@l-arginine	EtOH/reflux	10	95	This work

**Reaction conditions:** Reaction of benzaldehyde (1 mmol), dimedone (1 mmol), and malononitrile (1 mmol) g-C_3_N_4_@l-arginine (20 mg) and ethanol (7 mL) under reflux conditions.

**Table 7:** Comparison of catalytic activity of g-C_3_N_4_@l-arginine with other reported catalysts for the synthesis of 2,3-dihydro quinazoline derivatives^.^

Entry	Catalyst	Solvent/temperature	Time (min)	Quinazoline yield (%)	Ref
1	Titanium silicon oxide nanopowder	H_2_O/100 °C	120	94	^26^
2	Wang-OSO_3_H	H_2_O/100 °C	24	84	^22^
3	Y(NO_3_)_3_.6H_2_O	CH_3_CN	300	97	^27^
4	Montmorillonite-KSF	Solvent-free/100 °C	150	93	^41^
5	g-C_3_N_4_@l-arginine	EtOH/reflux	15	96	This work

Reaction condition: 4-chlorobenzaldehyde (1mmol), isotonic anhydride (1mmol), and ammonium acetate (1mmol), g-C_3_N_4_@l-arginine catalyst (20.00 mg), and ethanol (7 mL) under reflux.

## Experimental

### Reagents and apparatus

All chemicals were purchased from Merck and Sigma-Aldrich Co. Fourier Transform Infrared (FTIR) spectra were recorded on Tensor27. Nuclear Magnetic Resonance (NMR) data were acquired on a Varian-Inova 500 MHz. X-Ray Diffraction (XRD) patterns were obtained using Dron-8 diffractometer. Energy-dispersive X-ray (EDX) spectrum was recorded on Numerix DXP–X10P. Thermal gravimetric analysis (TGA) was performed using STA 504 instrument under argon atmosphere. Field Emission Scanning Electron Microscopy (FESEM) images were recorder with TESCAN-MIRA III.

### Preparation of bulk g-C3N4 and g-C3N4 nanosheets

For the synthesis of bulk g-C_3_N_4_, the melamine was heated at 550 °C in a furnace at the heating rate of 2.5 °C min^−1^ in static air for 4 h. A yellow powder was obtained which was then grounded in a ball mill. For the synthesis of g-C_3_N_4_ nanosheets, bulk g-C_3_N_4_ (1.0 g) was first stirred in H_2_SO_4_ (20 mL) at 90 °C for 5 h. The solution was then diluted with ethanol (200 mL) and stirred again at room temperature for 2 h. The resulting product was dispersed in 100.0 mL water/isopropanol (1:1) solution and sonicated for 6 h. Finally, the formed suspension was centrifuged at 5000 rpm to separate g-C_3_N_4_ nanosheets.

### Preparation of g-C3N4@l-arginine

g-C_3_N_4_ (1.0 g) nanosheets were dispersed in dry toluene (20.0 mL). Then, the reaction mixture was refluxed under N_2_ atmosphere for 24 h after addition of 1,3-dibromopropane (2.0 mL). Finally, the product was filtered and washed with ethyl acetate, and dried at room temperature. The resulting product was dissolved in a mixture of water and methanol (1:1) followed by the addition of l-arginine (1 mmol), K_2_CO_3_ (1.0 mmol), and NaI (1.0 mmol). The solution was stirred at room temperature for 24 h. the reaction mixture was then washed with water and methanol and dried at room temperature.

### General procedure for the synthesis of 1,4-dihydropyridine derivatives

A mixture of aldehyde (1.0 mmol), ethyl acetoacetate (1.0 mmol), dimedone (1.0 mmol), ammonium acetate (1.0 mmol), g-C_3_N_4_@l-arginine (20.0 mg), and ethanol (2.0 mL) was added in a round bottom flask and refluxed at 70 °C. When the reaction was completed (monitored by TLC), the catalyst was separated by filtration and washed with ethanol, and then to purifying the product was used recrystallization.

### General procedure for the synthesis of 2,3-dihydro quinazoline derivatives

In a round bottom flask, aldehyde (1.0 mmol), isotonic anhydride (1.0 mmol), ammonium acetate (2.0 mmol), and g-C_3_N_4_@l-arginine (20.0 mg) were added and refluxed in ethanol (2.0 mL) at 70 °C. After reaction completion (monitored by TLC), the catalyst was removed by filtration and washed with ethanol, and then to purifying the product was used recrystallization.

### General procedure for the synthesis of 4H-chromene derivatives

In a round bottom flask was added aldehyde (1.0 mmol), dimedone (1.0 mmol), malononitrile (1.0 mmol), g-C_3_N_4_@l-arginine (20.0 mg), and ethanol (2.0 mL). The mixture was then refluxed at 70 °C until the reaction was completed (monitored by TLC). At last, catalyst separation by filtration and washed with ethanol, and then to purifying the product was used recrystallization.

## Conclusions

In summary, heterogeneous g-C_3_N_4_@-arginine nanocatalyst was prepared and used for the synthesis of 1,4-dihydropyridine, 4H-chromene, and 2,3-dihydro quinazoline derivatives as important products in pharmacologically active compounds. The main advantages of this nanocatalyst is its reusability, simple separation from the reaction mixture, applicability for a broad range of high efficiency condensation reactions, and short reaction time. In addition, the use of an easy and convenient method for the preparation of the nanocatalyst is another advantage of this catalyst over other reported catalysts.

## Selected spectral data

### Ethyl 2,7,7-trimethyl-5-oxo-4-(4-hydroxylphenyl)-1,4,5,6,7,8-hexahydroquinoline-3-carboxylate (5c)

FTIR (KBr, cm-1): 3270, 3194, 3071, 2957, 1678, 1645, 1481, 1377, 1214 cm-1. 1H NMR (500 MHz, DMSO): δ H (ppm) = 0.85(s, 3H, CH3), 1.0(s, 3H, CH3), 1.13(t, 3H, CH3), 1.9–2.41(m,4H, 2CH2), 2.25(s, 3H, CH3), 3.95–3.99(q, 2H, OCH2), 4.73(s, 1H, Ar–CH), 6.54(d, 2H, Ar–H), 6.93(d, 2H, Ar–H), 8.95(s, 1H, NH), 9.01(s,1H, OH).

### Ethyl 1,4,7,8-tetrahydro-2,7,7-trimethyl-4-(4-nitrophenyl)-5(6H)-oxoquinoline-3-carboxylate (5d)

FTIR (KBr, cm-1): 3276, 3210, 3076, 2969, 2902, 1703, 1641, 1530, 1379 cm-1. 1H NMR (500 MHz, DMSO): δH (ppm) = 0.83(s, 3H, CH3), 1.01(s, 3H, CH3), 1.11(t, 3H, CH3), 1.96–2.46(m,4H, 2CH2), 2.31(s, 3H, CH3), 3.93–4.0(m, 2H, OCH2), 4.97(s, 1H, Ar–CH), 7.5–7.61 (m, 4H, Ar–H), 7.97(s, 1H, NH), 9.23(s,1H, OH).

### 2-amino-4-(4-nitrophenyl)-7,7-dimethyl-5-oxo-5,6,7,8-tetrahydro-4H-chromene-3-carbonitrile (9c)

FTIR(KBr, cm-1): 3403, 3312, 3170, 2969, 2881,2181, 1668, 1626, 1517, 1345, 856 cm-1.1 H NMR (500 MHz, DMSO): δ H (ppm) = 0.95(s, 3H, CH3), 1.04(s, 3H, CH3), 2.09–2.53(m, 4H, 2CH2), 4.36(s, 1H, CH), 7.1(s, 2H, NH2), 7.43–8.17(m, 4H, Ar–H).

### 2-phenyl-2, 3-dihydro-4(1H)-quinazolinone (13a)

FTIR (KBr, cm-1): 3300, 3176, 2981, 1651, 1610, 1507, 1440, 1385, 745 cm-1. 1H NMR (500 MHz, DMSO): δ H (ppm) = 5.75(s, 1H, CH), 6.67(t, 1H, Ar–H), 6.74(d, 1H, Ar–H), 7,1(s, 1H, NH), 7.23(t, 1H, Ar–H), 7.34(t, 1H, Ar–H), 7.38(t, 1H, Ar–H), 7.49(d, 1H, Ar–H), 7.60(d, 1H, Ar–H), 8.27(s, 1H, CONH).

### 2-(4-chloro-phenyl)-2, 3-dihydro-1H-quinazoline-4-one (13b)

FTIR (KBr, cm-1): 3305, 3184, 3062, 1654, 1606, 1431, 1090, 749 cm-1. 1H NMR (500 MHz, DMSO): δ H (ppm) = 5.77(s, 1H, CH), 6.68(t, 1H, Ar–H), 6.74(d, 1H, Ar–H), 7,1(s, 1H, NH), 7.24(t, 1H, Ar–H), 7.45(d, 1H, Ar–H), 7.50(d, 1H, Ar–H), 7.61(d, 1H, Ar–H), 8.27(s, 1H, CONH).

## Supplementary Information

Supplementary Information.
