# Supplementary material for: Preparation and characterization of graphitic carbon nitride-supported l-arginine as a highly efficient and recyclable catalyst for the one-pot synthesis of condensation reactions
Source: Sci Rep. 2021 Oct 5;11:19792. doi: 10.1038/s41598-021-97360-x (PMC8492782; doi:10.1038/s41598-021-97360-x)
Supplement: Supplementary file 1 — Supplementary Information. [file 41598_2021_97360_MOESM1_ESM.docx]

**Preparation and characterization of Graphitic Carbon Nitride - Supported L-arginine as a highly efficient and recyclable catalyst for the One-Pot Synthesis of Condensation Reactions**

Hossein Ghafuri ^*a^, Zeinab Tajik, Nastaran Ghanbari, Peyman Hanifehnejad

*Catalysts and Organic Synthesis Research Laboratory, Department of Chemistry, Iran University of Science and Technology, Tehran 16846‑13114, Iran.*

***Email:*** *ghafuri@iust.ac.ir*

| **Page** | **Content** |
| --- | --- |
| S1 | Title page |
| S2 | General procedure for preparation of the bulk g-C_3_N_4_ and g-C_3_N_4_ nanosheets |
| S2 | General procedure for preparation of the g-C_3_N_4_@L-arginine |
| S4 | Characterization of the g-C_3_N_4_@L-arginine |
| S9 | Chemical characterization of Chemical characterization Ethyl 2, 7, 7-trimethyl-5-oxo-4-(4-hydroxylphenyl)-1,4,5,6,7,8-hexahydroquinoline-3-carboxylate (**5c**) |
| S10 | Chemical characterization of Ethyl 1,4,7,8-tetrahydro-2,7,7-trimethyl-4-(4-nitrophenyl)-5(6H)-oxoquinoline-3-carboxylate (**5d**) |
| S12 | Chemical characterization of 2-phenyl-2, 3-dihydro-4(1H)-quinazolinone (**13a**) |
| S13 | Chemical characterization of 2-(4-chloro-phenyl)-2, 3-dihydro-1H-quinazoline-4-one (**13b**) |
| S15 | Chemical characterization of 2-amino-4-(4-nitrophenyl)-7,7-dimethyl-5-oxo-5,6,7,8-tetrahydro-4H-chromene-3-carbonitrile (**9c**) |

**Preparation of bulk g-C_3_N_4_ and g-C_3_N_4_ nanosheets**

For the synthesis of bulk g-C_3_N_4_, the melamine was heated at 550 ℃ temperature in a furnace at the ramp 2.5 ℃ min ^-1^ in static air for 4 h. A yellow powder was obtained, which was grounded with a ball mill. For the synthesis of g-C_3_N_4_ nanosheets, first bulk g-C_3_N_4_ (1.0 g) was stirred in H_2_SO_4_ (20 mL) at 90 ℃ for 5 h, then diluted with ethanol (200 mL) and was stirred again at room temperature for 2 h. The resulting product was dispersed in the 100.0 mL of water/isopropanol (1:1) and sonicated for 6 h. Finally, the formed suspension was centrifuged (5000 rpm) for g-C_3_N_4_ nanosheets separation.

**Preparation of g-C_3_N_4_@L-arginine**

Nanosheets g-C_3_N_4_ (1.0 g) were dispersed in dry toluene (20.0 mL) then, 1,3dibromopropane (2.0 mL) was added to the solution, and the reaction mixture was refluxed under N_2_ atmosphere for 24 h. Finally, the product was filtered and was washed with ethyl acetate, and dried at room temperature. The resulting product was solved in a mixture of water and methanol (1:1) then, L-arginine (1mmol), K_2_CO_3_ (1.0 mmol), and NaI (1.0 mmol) were added. The solution was stirred at room temperature for 24h; the reaction mixture was washed with water and methanol and dried at room temperature.


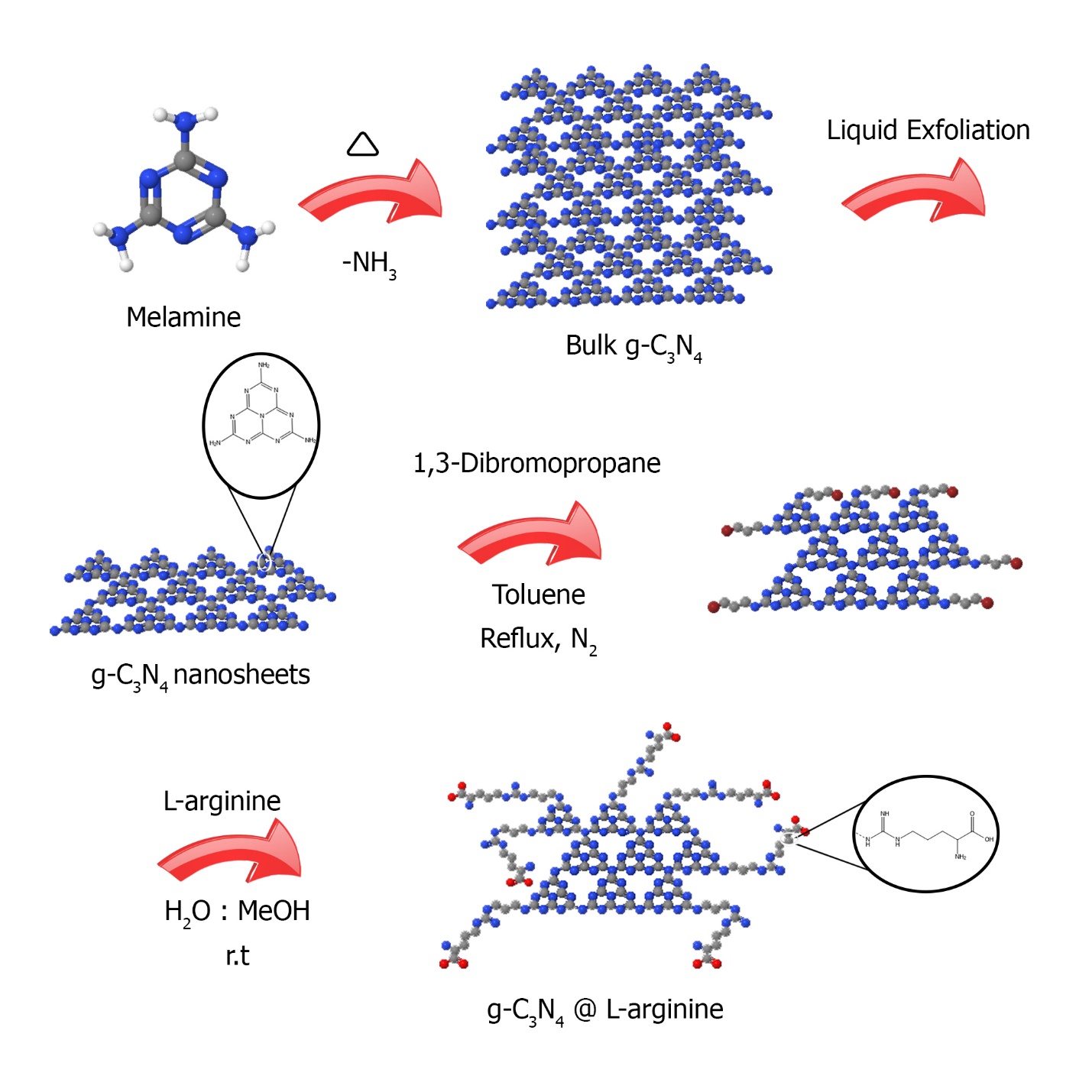


**Scheme S1.** Synthesis of g-C_3_N_4_@L-arginine

***Characterization of the g-C_3_N_4_@L-arginine***


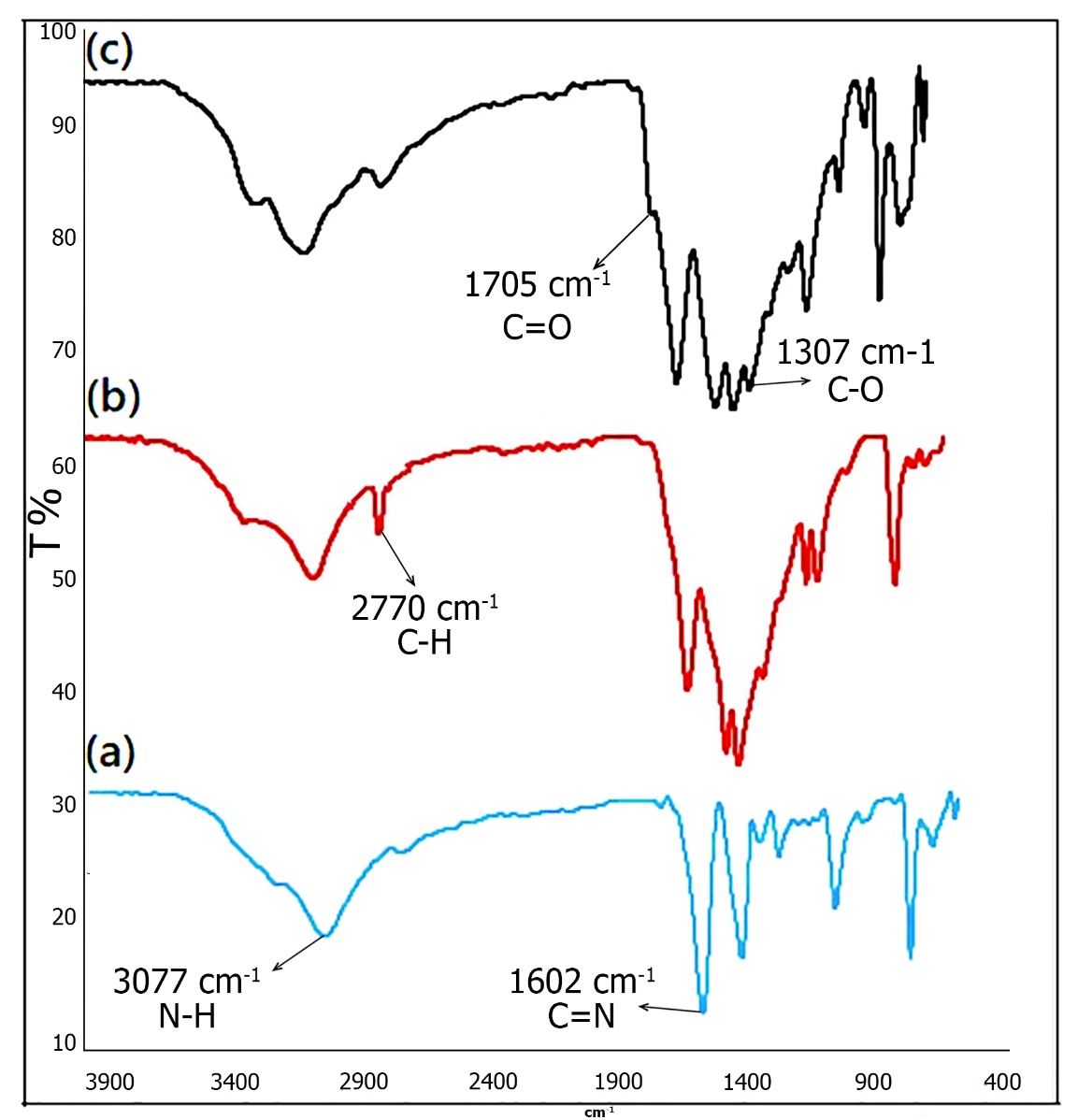


**Figure 1**. FT-IR spectra of (a) nanosheets g-C_3_N_4_, (b) modified g-C_3_N_4_, (c) g-C_3_N_4_@L-arginine.


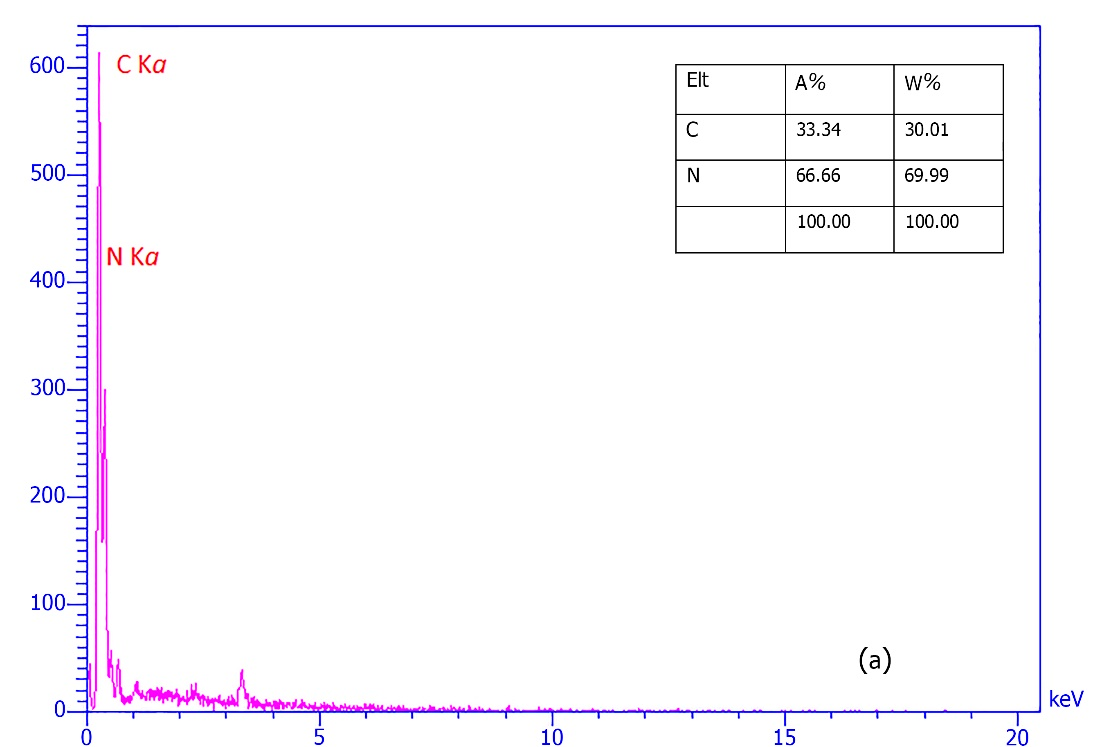


**
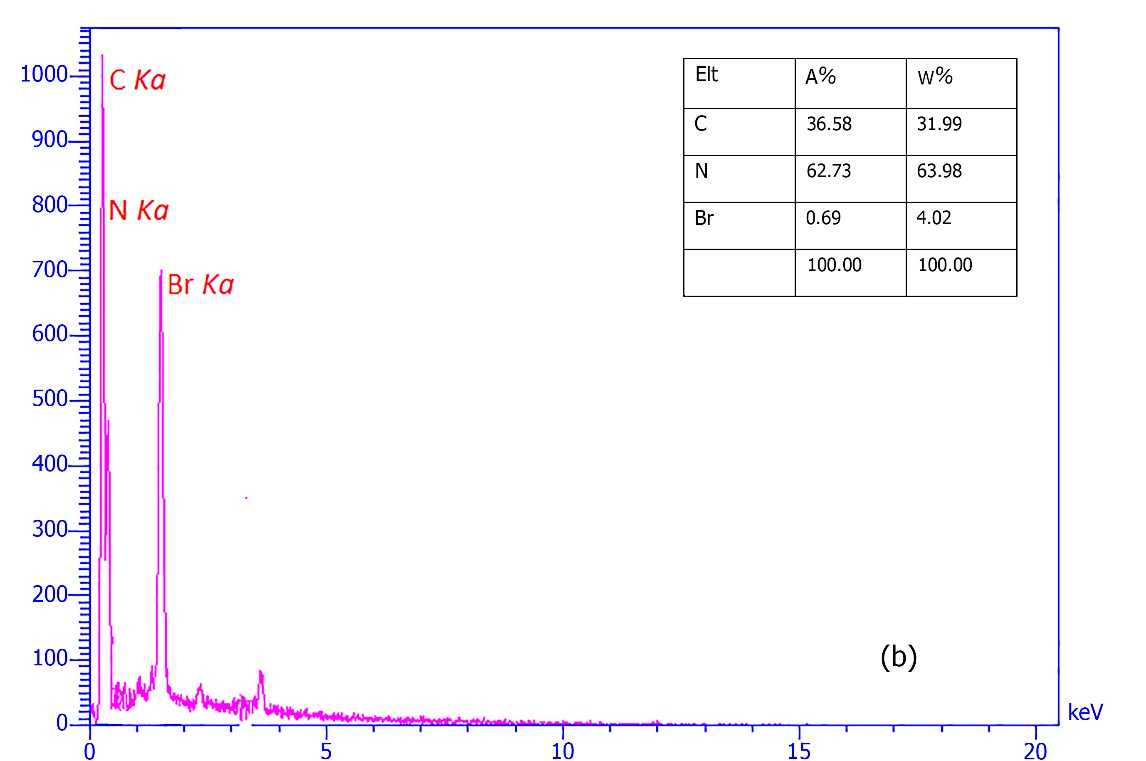
**

**
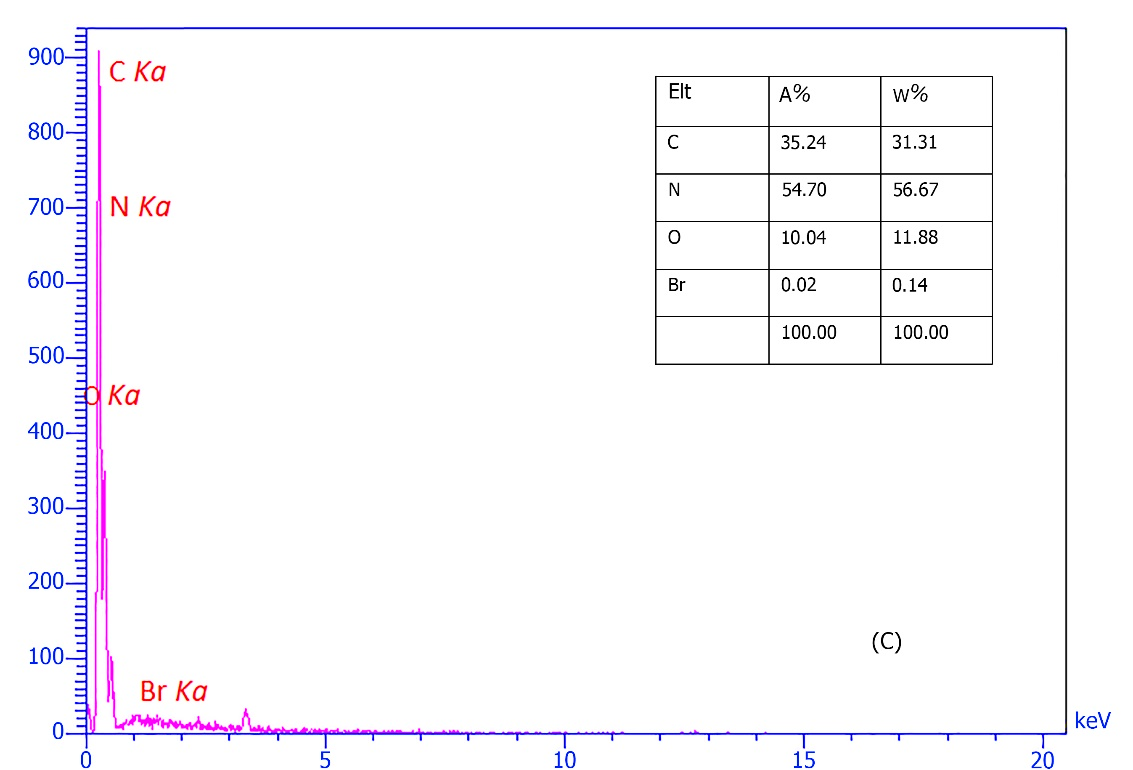
**

**Figure 2.** EDX spectrum of (a) nanosheets g-C_3_N_4_, (b) modified g-C_3_N_4_, (c) g-C_3_N_4_@L-arginine.


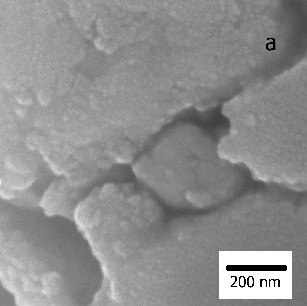
 **
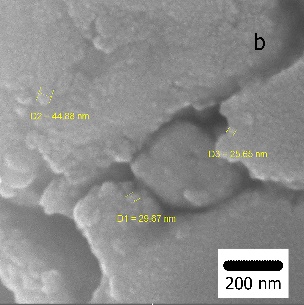
** **
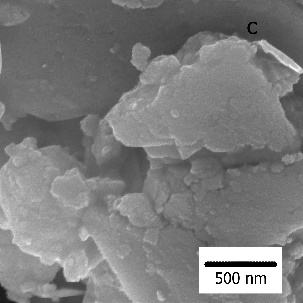

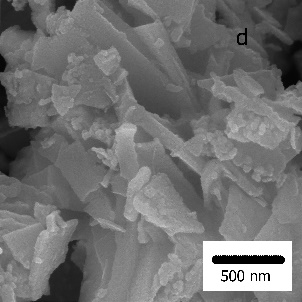
**

**
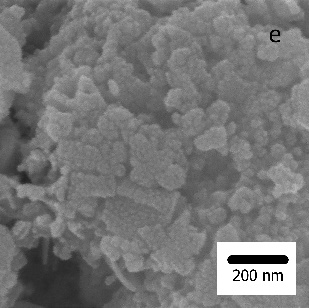
** **
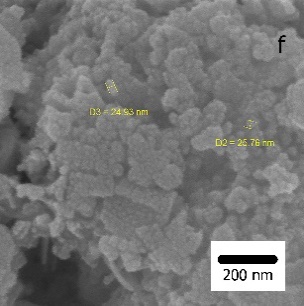
** **
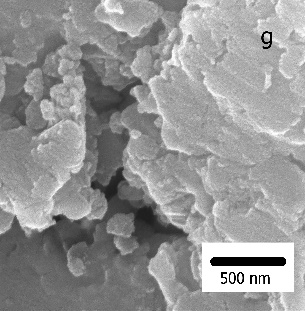
** **
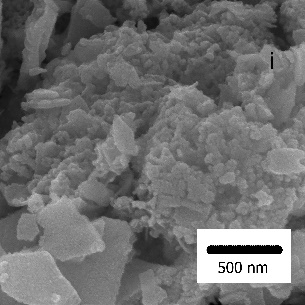
**

**Figure 3**. FE-SEM image of nanosheets g-C_3_N_4_ (a, b, c, and d), and g-C_3_N_4_@L-arginine (e, f, g, i).

**Figure 4.** XRD pattern of (a) nanosheets g-C_3_N_4_, (b) g-C_3_N_4_@L-arginine.


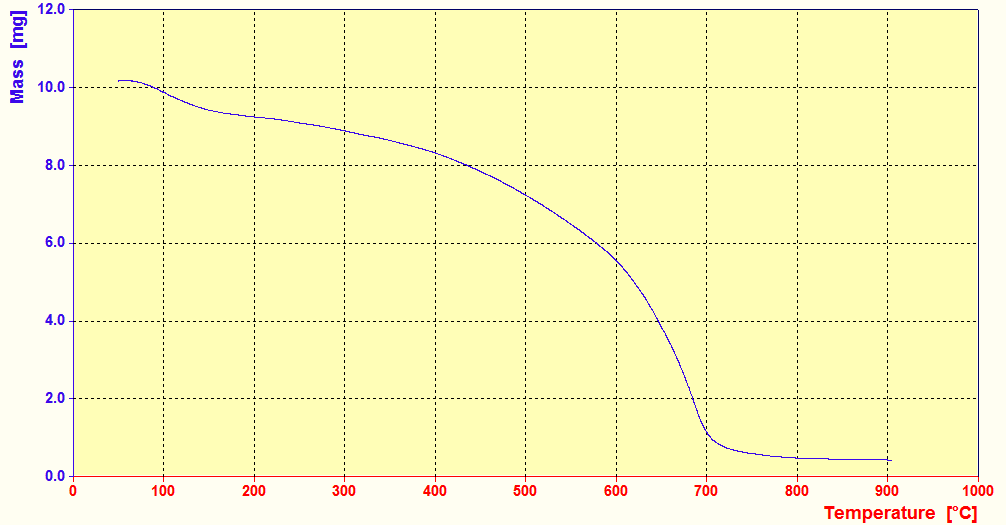


**Figure 5**. TGA analysis of g-C_3_N_4_@L-arginine.

**General procedure for the synthesis of 1, 4-dihydropyridine derivatives**

A mixture of aldehyde (1.0 mmol), ethyl acetoacetate (1.0 mmol), dimedone (1.0 mmol), ammonium acetate (1.0 mmol), g-C_3_N_4_@L-arginine (20.0 mg), and ethanol (2.0 mL) was added in a round bottom flask and refluxed at 70 ℃. When the reaction was completed (was monitored by TLC), the catalyst was separated by filtration.

**General procedure for the synthesis of 2,3-dihydro quinazoline derivatives**

In a round bottom flask, aldehyde (1.0 mmol), isotonic anhydride (1.0 mmol), ammonium acetate (2.0 mmol), and g-C_3_N_4_@L-arginine (20.0 mg) were added and refluxed in ethanol (2.0 mL) at 70℃. After completion of the reaction (was monitored by TLC), the catalyst was removed by filtration and washed with ethanol.

**General procedure for the synthesis of 4*H*-chromene derivatives**

In a usual experiment, in a round bottom flask were added aldehyde (1.0 mmol), dimedone (1.0 mmol), malononitrile (1.0 mmol), g-C_3_N_4_@L-arginine (20.0 mg), and ethanol (2.0 mL). Then refluxed at 70 ℃ until the reaction was completed (was monitored by TLC), at last, catalyst separation and crystallization was performed as above.

**1. Selected Spectral Data:**

- 1. **Ethyl 2, 7, 7-trimethyl-5-oxo-4-(4-hydroxylphenyl)-1,4,5,6,7,8-hexahydroquinoline-3-carboxylate (5c)**

FTIR (KBr, cm-1): 3270, 3194, 3071, 2957, 1678, 1645, 1481, 1377, 1214 cm-1. 1H NMR (500 MHz, DMSO): δ H (ppm)= 0.85(s, 3H, CH3), 1.0(s, 3H, CH3), 1.13(t, 3H, CH3), 1.9-2.41(m,4H, 2CH2), 2.25(s, 3H, CH3), 3.95-3.99(q, 2H, OCH2), 4.73(s, 1H, Ar-CH), 6.54(d, 2H, Ar-H), 6.93(d, 2H, Ar-H), 8.95(s, 1H, NH), 9.01(s,1H, OH).


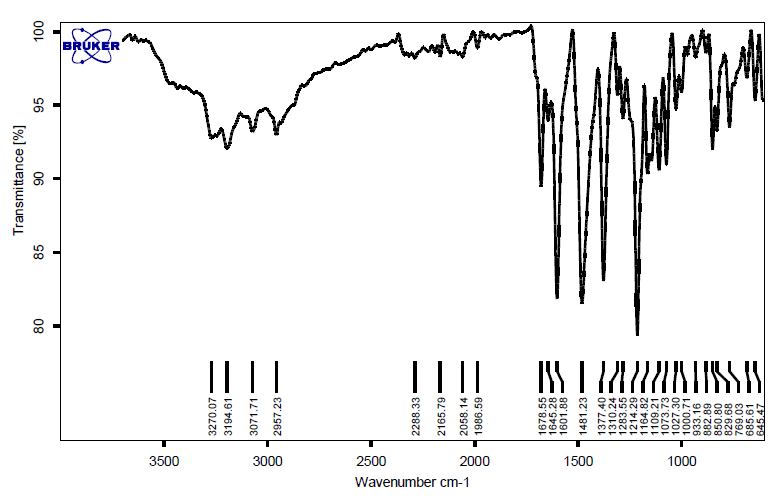


**Fig. S1**. FT-IR spectrum of the Ethyl 2, 7, 7-trimethyl-5-oxo-4-(4-hydroxylphenyl)-1,4,5,6,7,8-hexahydroquinoline-3-carboxylate (5c)


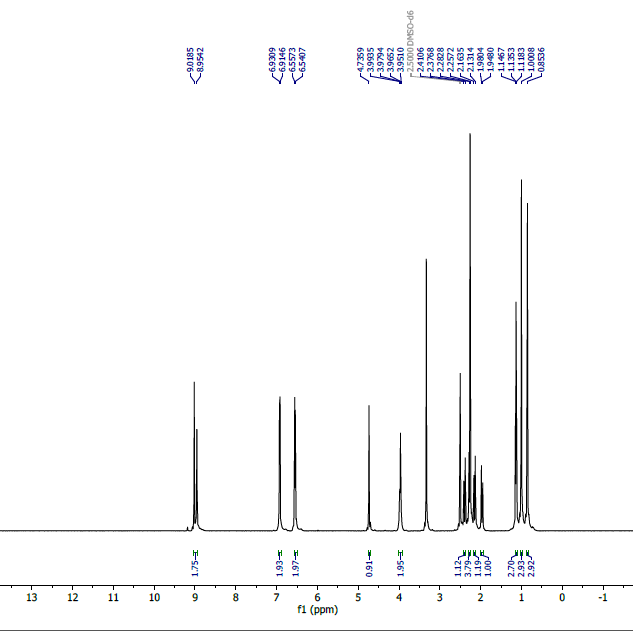


**Fig. S2.** ^1^HNMR spectrum of the Ethyl 2, 7, 7-trimethyl-5-oxo-4-(4-hydroxylphenyl)-1,4,5,6,7,8-hexahydroquinoline-3-carboxylate (5c)

- 1. **Ethyl 1,4,7,8-tetrahydro-2,7,7-trimethyl-4-(4-nitrophenyl)-5(6H)-oxoquinoline-3-carboxylate (5d)**

FTIR (KBr, cm-1): 3276, 3210, 3076, 2969, 2902, 1703, 1641, 1530, 1379 cm-1. 1H NMR (500 MHz, DMSO): δH (ppm)= 0.83(s, 3H, CH3), 1.01(s, 3H, CH3), 1.11(t, 3H, CH3), 1.96-2.46(m,4H, 2CH2), 2.31(s, 3H, CH3), 3.93-4.0(m, 2H, OCH2), 4.97(s, 1H, Ar-CH), 7.5-7.61 (m, 4H, Ar-H), 7.97(s, 1H, NH), 9.23(s,1H, OH).


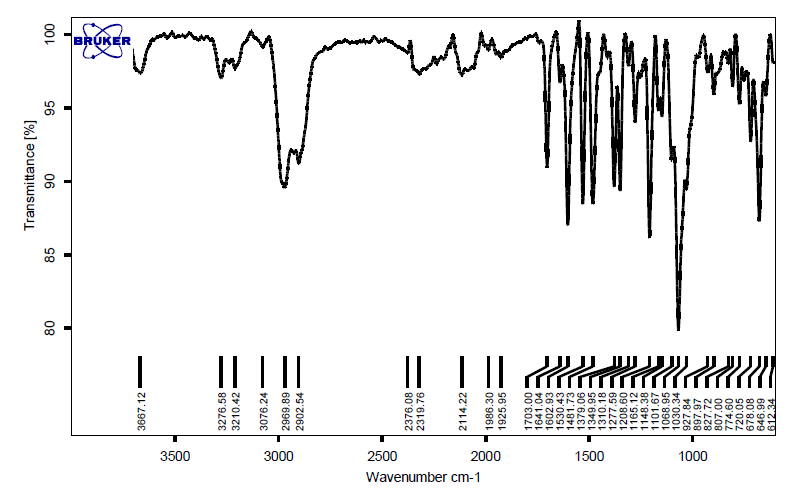


**Fig. S3**. FT-IR spectrum of the Ethyl 1,4,7,8-tetrahydro-2,7,7-trimethyl-4-(4-nitrophenyl)-5(6H)-oxoquinoline-3-carboxylate (5d)


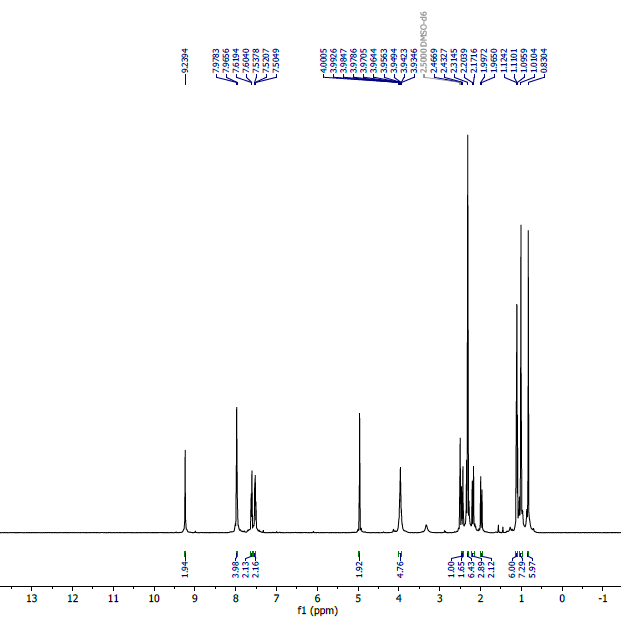


**Fig. S4.** ^1^HNMR spectrum of the Ethyl 1,4,7,8-tetrahydro-2,7,7-trimethyl-4-(4-nitrophenyl)-5(6H)-oxoquinoline-3-carboxylate (5d)

- 1. **2-phenyl-2, 3-dihydro-4(1H)-quinazolinone (13a)**

FTIR (KBr, cm-1): 3300, 3176, 2981, 1651, 1610, 1507, 1440, 1385, 745 cm-1. 1H NMR (500 MHz, DMSO): δ H (ppm)= 5.75(s, 1H, CH), 6.67(t, 1H, Ar-H), 6.74(d, 1H, Ar-H), 7,1(s, 1H, NH), 7.23(t, 1H, Ar-H), 7.34(t, 1H, Ar-H), 7.38(t, 1H, Ar-H), 7.49(d, 1H, Ar-H), 7.60(d, 1H, Ar-H), 8.27(s, 1H, CONH).


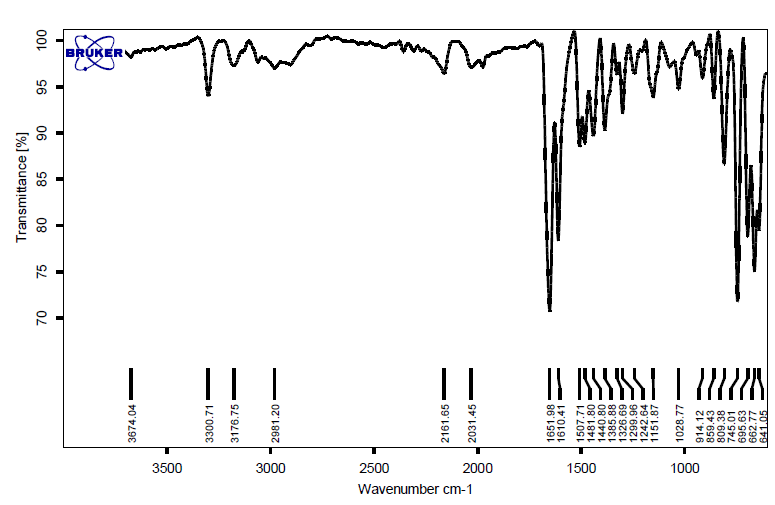


**Fig. S5.** ^1^HNMR spectrum of the 2-phenyl-2, 3-dihydro-4(1H)-quinazolinone (13a)


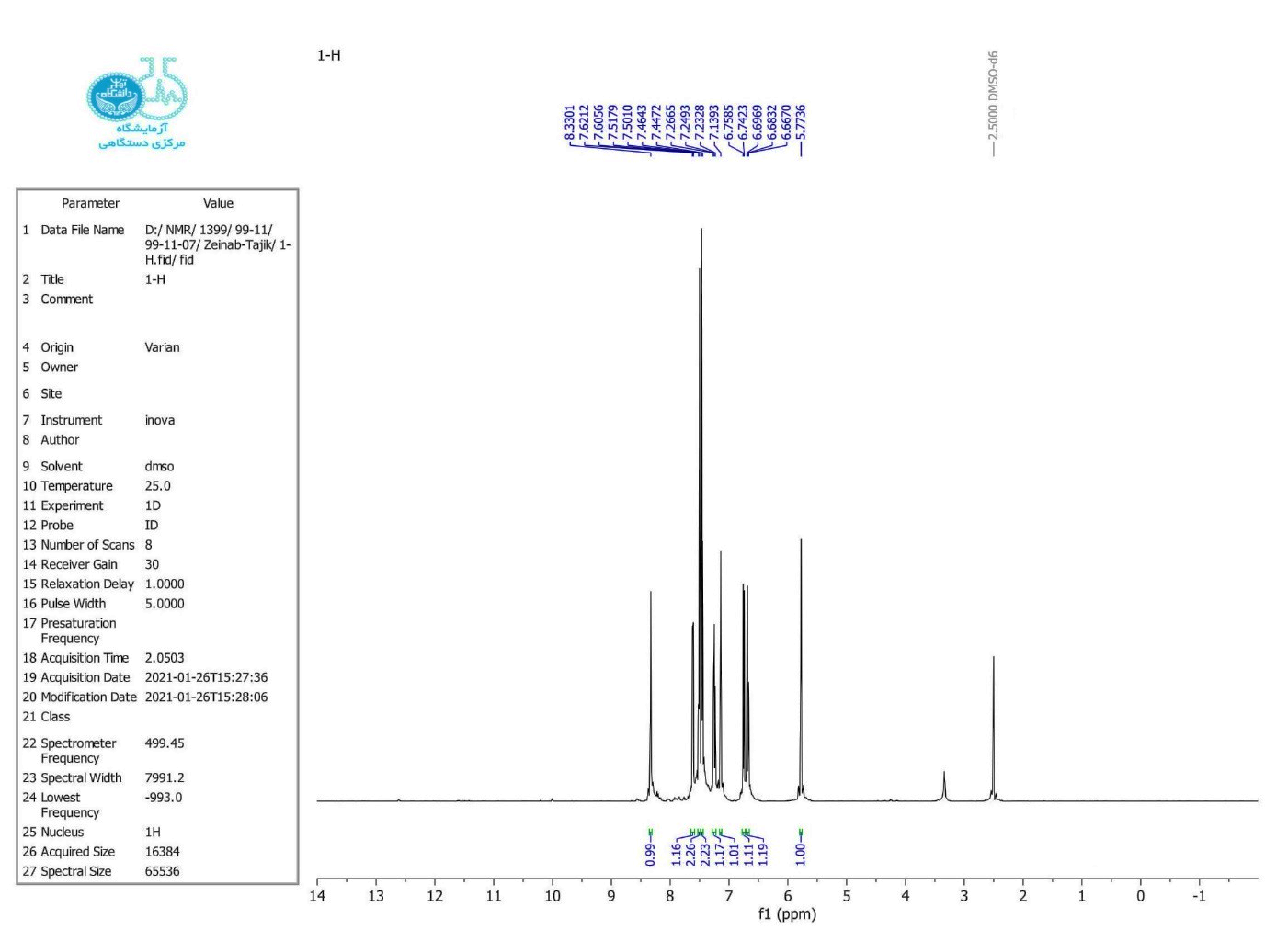


**Fig. S6.** 1HNMR spectrum of the 2-phenyl-2, 3-dihydro-4(1H)-quinazolinone (13a)

- 1. **2-(4-chloro-phenyl)-2, 3-dihydro-1*H*-quinazoline-4-one (13b)**

FTIR (KBr, cm^-1^): 3305, 3184, 3062, 1654, 1606, 1431, 1090, 749 cm^-1^. ^1^H NMR (500 MHz, DMSO): **δ** H (ppm)= 5.77(s, 1H, CH), 6.68(t, 1H, Ar-H), 6.74(d, 1H, Ar-H), 7,1(s, 1H, NH), 7.24(t, 1H, Ar-H), 7.45(d, 1H, Ar-H), 7.50(d, 1H, Ar-H), 7.61(d, 1H, Ar-H), 8.27(s, 1H, CONH).


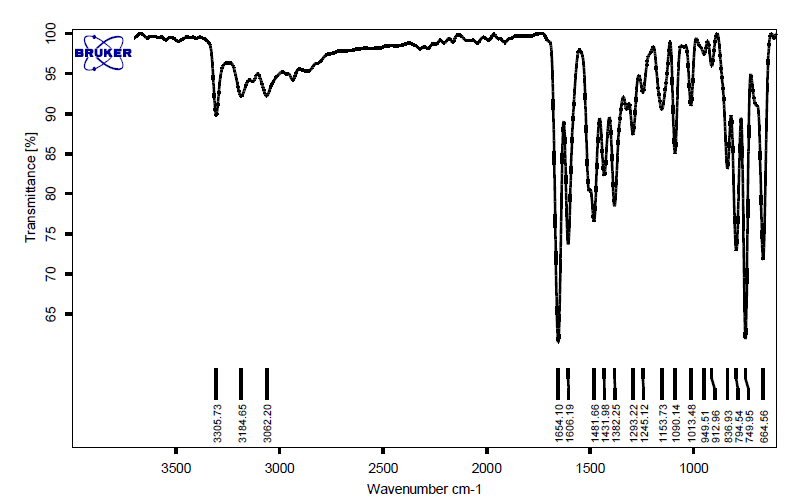


**Fig. S7.** 1HNMR spectrum of the 2-(4-chloro-phenyl)-2, 3-dihydro-1H-quinazoline-4-one (13b)


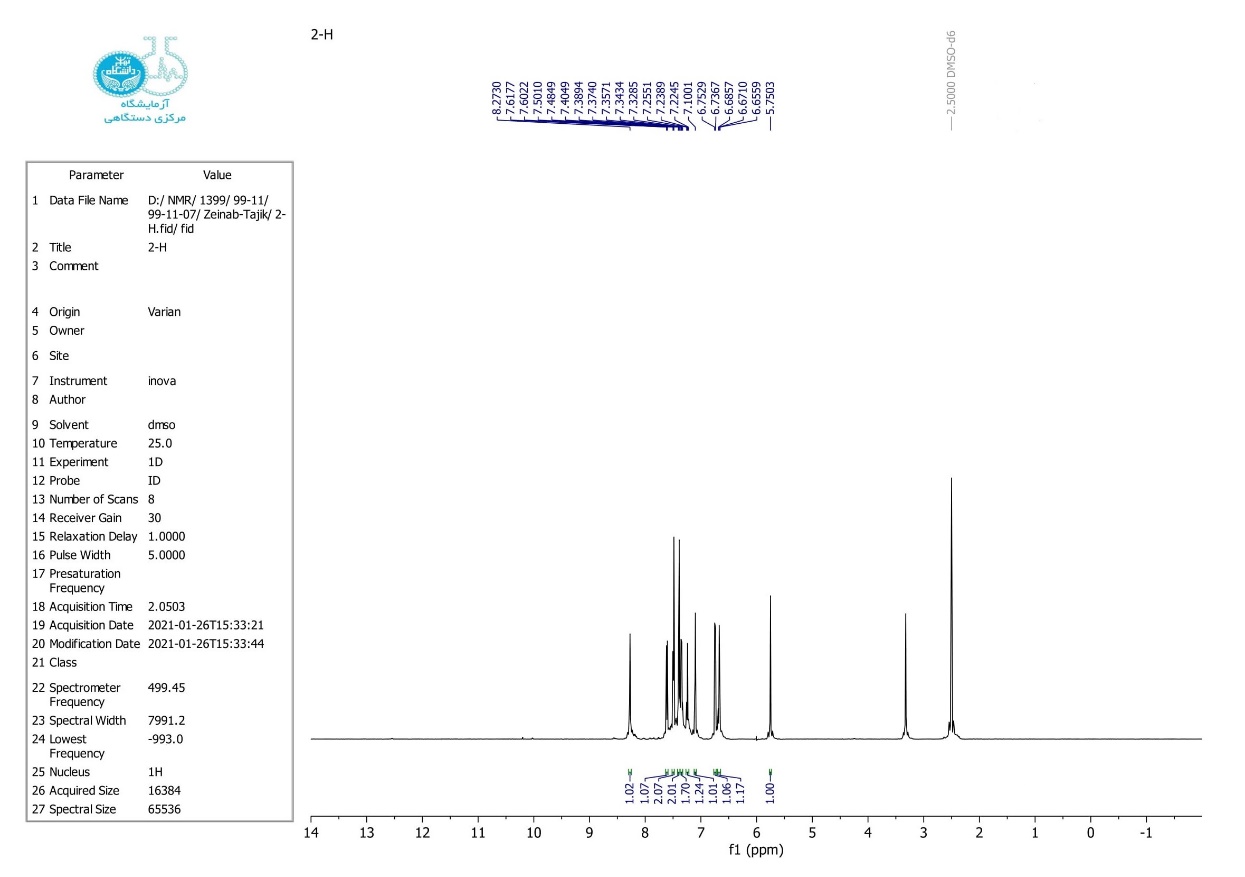


**Fig. S7.** 1HNMR spectrum of the 2-(4-chloro-phenyl)-2, 3-dihydro-1H-quinazoline-4-one (13b)

- 1. **2-amino-4-(4-nitrophenyl)-7,7-dimethyl-5-oxo-5,6,7,8-tetrahydro-4*H*-chromene-3-carbonitrile (9c)**

FTIR (KBr, cm-1): 3403, 3312, 3170, 2969, 2881,2181, 1668, 1626, 1517, 1345, 856 cm-1.1 H NMR (500MHz, DMSO): δ H (ppm)= 0.95(s, 3H, CH3), 1.04(s, 3H, CH3), 2.09-2.53(m, 4H, 2CH2), 4.36(s, 1H, CH), 7.1(s, 2H, NH2), 7.43-8.17(m, 4H, Ar-H).


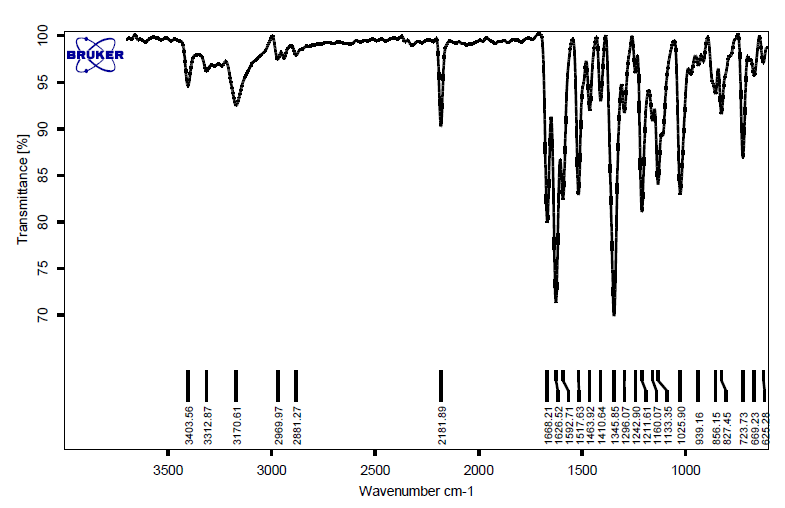


**Fig. S8.** 1HNMR spectrum of the 2-amino-4-(4-nitrophenyl)-7,7-dimethyl-5-oxo-5,6,7,8-tetrahydro-4H-chromene-3-carbonitrile (9c)


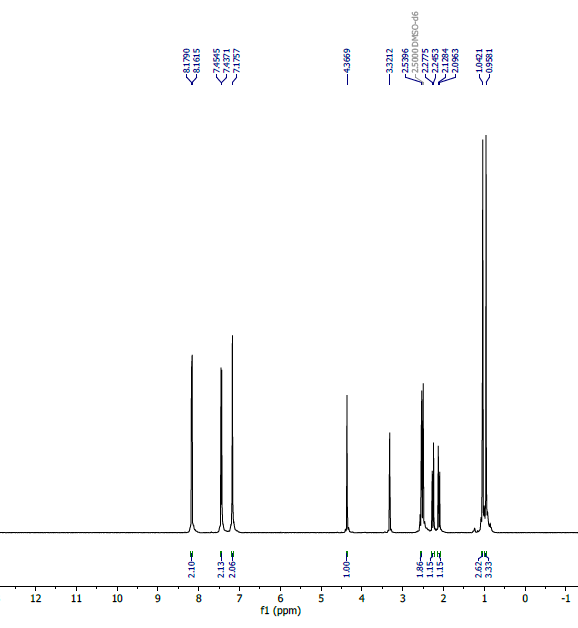


**Fig. S9.** 1HNMR spectrum of the 2-amino-4-(4-nitrophenyl)-7,7-dimethyl-5-oxo-5,6,7,8-tetrahydro-4H-chromene-3-carbonitrile (9c)
